# OLGenie: Estimating Natural Selection to Predict Functional Overlapping Genes

**DOI:** 10.1093/molbev/msaa087

**Published:** 2020-04-03

**Authors:** Chase W Nelson, Zachary Ardern, Xinzhu Wei

**Affiliations:** m1 Sackler Institute for Comparative Genomics, American Museum of Natural History, New York, NY; m2 Biodiversity Research Center, Academia Sinica, Taipei, Taiwan; m3 Microbial Ecology, ZIEL—Institute for Food & Health, Technische Universität München, Freising, Germany; m4 Department of Ecology and Evolutionary Biology, University of Michigan, Ann Arbor, MI; m5 Department of Integrative Biology and Statistics, University of California, Berkeley, CA

**Keywords:** *antisense protein* (*asp*) gene, *d*_N_/*d*_S_, gene prediction, genome annotation, human immunodeficiency virus-1, open reading frame, overlapping gene (OLG), purifying (negative) selection

## Abstract

Purifying (negative) natural selection is a hallmark of functional biological sequences, and can be detected in protein-coding genes using the ratio of nonsynonymous to synonymous substitutions per site (*d*_N_/*d*_S_). However, when two genes overlap the same nucleotide sites in different frames, synonymous changes in one gene may be nonsynonymous in the other, perturbing *d*_N_/*d*_S_. Thus, scalable methods are needed to estimate functional constraint specifically for overlapping genes (OLGs). We propose OLGenie, which implements a modification of the Wei–Zhang method. Assessment with simulations and controls from viral genomes (58 OLGs and 176 non-OLGs) demonstrates low false-positive rates and good discriminatory ability in differentiating true OLGs from non-OLGs. We also apply OLGenie to the unresolved case of HIV-1’s putative *antisense protein* gene, showing significant purifying selection. OLGenie can be used to study known OLGs and to predict new OLGs in genome annotation. Software and example data are freely available at https://github.com/chasewnelson/OLGenie (last accessed April 10, 2020).

Natural selection in protein-coding genes is commonly inferred by comparing the number of nonsynonymous (amino acid changing; *d*_N_) and synonymous (not amino acid changing; *d*_S_) substitutions per site, with *d*_N_/*d*_S_ <1 indicative of purifying (negative) selection. Thus, *d*_N_/*d*_S_ can be used to predict functional genes (Gojobori et al. 1982; Nekrutenko et al. 2002). However, complications arise if synonymous changes are not neutral, in which case purifying selection may reduce *d*_S_ (i.e., increase *d*_N_/*d*_S_). This is usually negligible, as the effects of most synonymous variants are dwarfed by those of disadvantageous nonsynonymous variants, causing the majority of genes to exhibit *d*_N_/*d*_S_ <1 (Hughes 1999; Holmes 2009). However, this assumption does not hold for overlapping genes (OLGs). A double-stranded nucleic acid may encode up to six open reading frames (ORFs), three in the sense direction and three in the antisense direction, allowing pairs of genes to overlap the same nucleotide positions in a genome (fig. 1). In such OLGs, changes that are synonymous in one gene may be nonsynonymous in the other, making otherwise “silent” variants subject to selection. As a result, *d*_N_/*d*_S_ methods designed for regular (non-overlapping) genes do not take into account the nonsynonymous effects (in the alternate gene) of some synonymous changes (in the reference gene). As a result, standard (non-OLG) *d*_N_/*d*_S_ methods can fail to detect purifying selection or erroneously predict positive (Darwinian) selection when applied to OLGs (Holmes et al. 2006; Sabath et al. 2008; Sabath and Graur 2010).

**Fig. 1:**
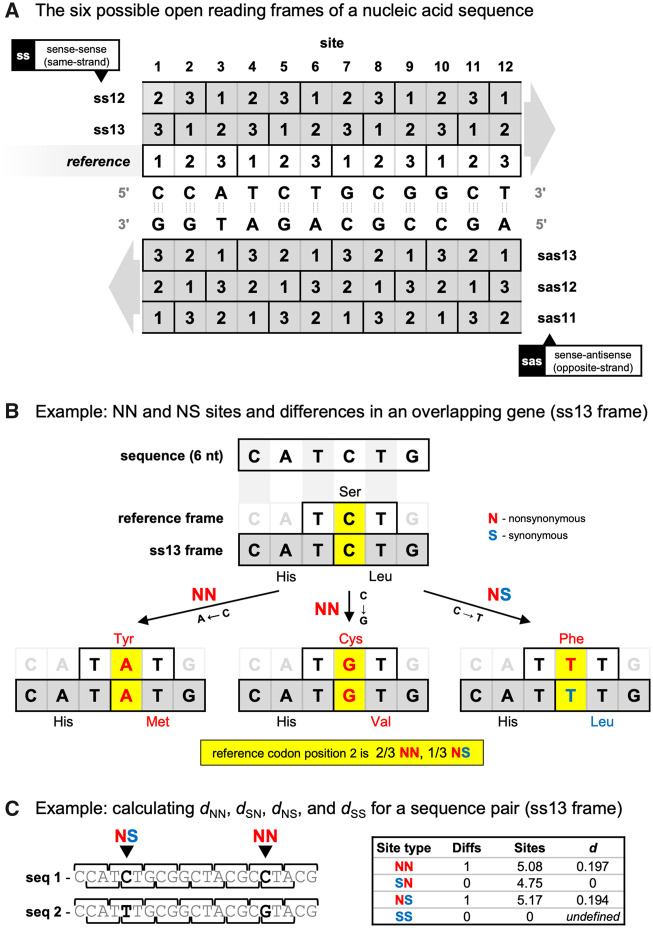
Overlapping genes: reading frames and terminology. (*A*) The six possible protein-coding open reading frames (ORFs) of a double-stranded nucleic acid sequence. Codons are denoted with solid black boxes, each comprising three ordered nucleotide positions (1, 2, 3) with light gray boundaries. The reference gene frame is shown with a white background, whereas alternate gene frames are shown with a gray background. Frame relationships are indicated using the nomenclature of Wei and Zhang (2015), where “ss” indicates “sense–sense” (same-strand), “sas” indicates “sense–antisense” (opposite-strand), and the numbers indicate which codon position of the alternate gene (second number) overlaps codon position 1 of the reference gene (first number). For all alternate frames except sas13, one reference codon partially overlaps each of two alternate codons. (*B*) Example of an overlapping gene in the ss13 frame. A minimal overlapping unit of 6 nt is shown, comprising one reference gene codon and its two overlapping codons in the alternate gene. At position 2 of the reference codon (highlighted in yellow), three nucleotide changes are possible: two cause nonsynonymous changes in both genes (NN; nonsynonymous/nonsynonymous) and one causes a nonsynonymous change in the reference gene but a synonymous change in the alternate gene (NS; nonsynonymous/synonymous). No synonymous/nonsynonymous (SN) or synonymous/synonymous (SS) changes are possible at this site. Thus, this site is counted as two-thirds of an NN site and one-third of an NS site. Finally, a pair of sequences having a C/A or C/G difference at this site is counted as having 1 NN difference, whereas a pair of sequences having a C/T difference at this site is counted as having 1 NS difference. (*C*) Example calculation of *d*_NN_, *d*_SN_, *d*_NS_, and *d*_SS_ for a pair of sequences with an overlapping gene in ss13. Codons are denoted with brackets above (reference gene) and below (alternate gene) each sequence. The distance *d* is calculated for each site type (NN, SN, NS, and SS) as the number of differences divided by the number of sites of that type. Because the first and last reference codons only partially overlap alternate codons, they are excluded from analysis and the numbers of sites sum to 15 (= 5 codons × 3 nt; codons 2–6). Numbers of sites are not an exact multiple of 1/3 because nucleotide 6 of sequence 2 (TTT; alternate codon TTG) does not tolerate a change to A, as this would lead to a stop codon in the alternate gene (TAG). Thus, this position is considered an SN site in sequence 1, but one-half of an NN site and one-half of an SN site in sequence 2, for a mean of 0.25 NN and 0.75 SN sites. The table shows the mean numbers of sites for the two sequences (sequence 1 = 4.33 NN, 5 SN, 5.67 NS, and 0 SS; sequence 2 = 5.83 NN, 4.5 SN, 4.67 NS, and 0 SS), used to calculate each *d* value. For a multiple sequence alignment, the mean number of differences and sites for all pairwise comparisons would be used.

OLGs are widespread in viruses (Belshaw et al. 2007; Brandes and Linial 2016; Pavesi et al. 2018), and may not be uncommon in prokaryotes (Meydan et al. 2018; Vanderhaeghen et al. 2018; Weaver et al. 2019) and eukaryotes, including humans (Makałowska et al. 2007; Sanna et al. 2008). The number of OLGs has likely been underestimated, partly because genome annotation software is biased against both short ORFs (Warren et al. 2010) and overlapping ORFs (Vanderhaeghen et al. 2018). Current methods for detecting OLGs, such as Synplot2 (Firth 2014), *d*_N_/*d*_S_ estimators (Sabath et al. 2008; Wei and Zhang 2015), and long-ORF identifiers (Schlub et al. 2018) are subject to one or more of the following limitations: restricted to long OLGs, limited to single or pairs of sequences, unsuitable for low sequence divergence, not specific to protein-coding genes, lacking accessible implementation, or too computationally intensive for genome-scale data (Table 1). For example, those available methods that are suitable for genome-scale analysis are not able to specifically detect protein-coding OLGs. Scalable bioinformatics tools are therefore needed to predict OLG candidates for further analysis, preferably by utilizing the evolutionary information available in multiple sequences and quantifying purifying selection in a way that is comparable with that of non-OLGs. We wrote OLGenie to fill this void.

**Table 1:** Methods with Available Implementations for Detecting Selection in Overlapping Genes.

Program^a^	Reference	Target	Implementation	Method Description	Advantages and Limitations	Available from
OLGenie	This study	Protein-coding sequence	Perl	Estimates *d*_N_/*d*_S_ by introducing three modifications to Wei–Zhang: 1) minimal overlapping units of 6 nt, that is, 1 reference codon and 2 alternate codons; 2) the Nei–Gojobori method; and 3) only single nucleotide differences rather than all mutational pathways	Fast; applicable to multiple sequence alignments; tree-agnostic; conservative for purifying selection and high levels of divergence, but nonconservative for positive selection; loss of power for pairwise distance >0.1 and neighboring variants	https://github.com/chasewnelson/OLGenie, last accessed April 10, 2020.
“Frameshift”	Schlub et al. (2018)	Protein-coding sequence	R	Finds ORFs longer than expected by chance given nucleotide context; includes two complementary methods: “codon permutation” and “synonymous mutation”	Medium to high accessibility as an R script requiring minor modifications. Can only detect relatively long OLGs. Slow for long sequences.	https://github.com/TimSchlub/Frameshift, last accessed April 10, 2020.
“StopStatistics”	Cassan et al. (2016)	Protein-coding sequence	Python, bash	Tests for depletion of those stop codons in sas12 that would be synonymous in reference; also applicable to enrichment of start codons	Low accessibility; scripts specific to particular data sets	https://figshare.com/s/9668ef62e84488d4787a, last accessed April 10, 2020.
FRESCo	Sealfon et al. (2015)	Constraint at synonymous sites	HYPHY batch language	Rates of nucleotide evolution across an alignment inferred using a maximum-likelihood model. Models of neutral and nonneutral evolution tested in sliding windows to infer regions with excess synonymous constraint	Suitable for short genomes/regions despite using a codon model; requires a phylogenetic tree; performs best at deep sequence coverage and increased sequence divergence	https://static-content.springer.com/esm/art%3A10.1186%2Fs13059-015-0603-7/MediaObjects/13059_2015_603_MOESM1_ESM.zip, last accessed April 10, 2020.
Wei–Zhang method	Wei and Zhang (2015)	Protein-coding sequence	Perl	Estimates *d*_N_/*d*_S_ in minimal-length coding regions flanked by variant-free codons (i.e., data-dependent minimal overlapping units) to determine the effects of all mutational pathways in the reference and alternate genes using the modified Nei–Gojobori method	Accurate but slow, especially for highly diverged sequences; tree-agnostic; outperforms Sabath et al. method (according to Wei and Zhang [2015]); only implemented for pairs of sequences; low accessibility and scalability	http://www.umich.edu/~zhanglab/download/Xinzhu_GBE2014/index.htm, last accessed April 10, 2020.
Synplot2	Firth (2014)	Constraint at synonymous sites	C++; Web-interface	Evolution at synonymous sites in a codon alignment compared to a null model of neutral evolution in order to infer sites with excess constraint; expected diversity at synonymous sites is set equal to diversity over the full alignment, and diversity is measured between sequential pairs around a phylogenetic tree	Medium accessibility; fast; limited use in the case of sas12; requires a phylogenetic tree; does not distinguish between coding and noncoding overlapping features	http://guinevere.otago.ac.nz/cgi-bin/aef/synplot.pl, last accessed April 10, 2020
						http://www.firthlab.path.cam.ac.uk/SynPlot2.zip, last accessed April 10, 2020.
KaKi (“Multilayer”)	Rubinstein et al. (2011)	Unexpected variation at synonymous sites	C++	Maximum-likelihood codon model approach that allows variation in both the synonymous and nonsynonymous substitution rates along a sequence; accounting for variability in the baseline substitution rate allows more reliable inference of positive selection	Low accessibility (requires an old Linux distribution to install); requires a phylogenetic tree; complex input and results; focus of explicit testing is on positive selection; applicable (but not specific) to protein-coding OLGs.	https://www.tau.ac.il/~talp/multilayer.tar.gz, last accessed April 10, 2020.
						https://www.tau.ac.il/~talp/readme.txt, last accessed April 10, 2020.
Sabath et al. method	Sabath et al. (2008)	Protein-coding sequence	MATLAB	Maximum-likelihood framework for estimating *d*_N_/*d*_S_; similar to the (nonimplemented) method of Pedersen and Jensen (2001)	Slower than Wei–Zhang; not recommended for highly similar sequences (pairwise distance <0.08); similar to OLGenie in the use of 6 nt (“sextet”) units; only implemented for pairs of sequences; low accessibility and scalability	http://nsmn1.uh.edu/dgraur/Software.html, last accessed April 10, 2020.
MLOGD	Firth and Brown (2006)	Protein-coding sequence	C++	Simple statistics on properties of sequence variation by codon position, and a maximum-likelihood statistic (MLOGD) taking into account nucleotide and amino acid substitution rates and codon usage	Less sensitive at detecting OLGs than Synplot2 (according to Firth [2014]); requires a minimum of ∼20 independent nucleotide variants; sas12 frame generates false-positives.	http://guinevere.otago.ac.nz/aef/MLOGD/software.html, last accessed April 10, 2020.

a Programs in descending order by year of publication; methods lacking implementations at active URLs are not listed.

## New Approaches

OLGenie is executed at the Unix/Linux command line with two inputs: 1) a multiple sequence alignment (FASTA file) of contiguous codons known or hypothesized to constitute an OLG pair; and 2) the frame relationship of the OLGs. The codon frame beginning at site 1 of the alignment is considered the “reference” gene, which overlaps one “alternate” gene. The choice of which gene to consider the reference versus the alternate is arbitrary; however, in practice, the reference gene ORF is typically longer, whereas the alternate gene ORF usually occurs entirely or partially within the reference gene, and is of unknown or more recently established functionality (Pavesi et al. 2018). The alternate gene can occur in any one of five frames: ss12, ss13, sas11, sas12, or sas13. Here, “ss” indicates “sense–sense” (same-strand), “sas” indicates “sense–antisense” (opposite-strand), and the numbers indicate which codon position of the alternate gene (second number) overlaps codon position 1 of the reference gene (first number) (fig. 1). We prefer this nomenclature because the meaning of each frame is described in its name; however, at least nine others have been employed, summarized in Table 2.

**Table 2:** Nomenclature for Overlapping Protein-Coding Reading Frames.

Study^a^	Frame^b^					
	5′- **123123** -3′	5′- **123 ** -3′	5′- **123 ** - 3′	5′- **123 ** -3′	5′- **123 ** -3′	5′- **123123** -3′
	5′- **123123** -3′	5′- **123123** -3′	5′- **123123** -3′	3′- **321321** -5′	3′- **321321** -5′	3′- **321321** -5′
OLGenie; Wei and Zhang (2015)	Reference (ss11)	ss12	ss13	sas11	sas12	sas13
Scherer et al. (2018)	+1	+3	+2	−3	−2	−1
Lèbre and Gascuel (2017)	+0	+2	+1	−1	−2	−0
Schlub et al. (2018)	+0	+2	+1	−c2	−c1	−c0
Sabath et al. (2008)	0	2 (same-strand)	1 (same-strand)	1 (opposite-strand)	2 (opposite-strand)	0 (opposite-strand)
Belshaw et al. (2007)	0	−1	+1	rc-1	rc+1	rc0
Firth and Brown (2006) ^c^	0	+2	+1	−1	−2	−3
Rogozin et al. (2002) ^c^	−	−	−	C1	C3	C2
Krakauer (2000) ^c^	−	+2	+1	−1	0	−2
Smith and Waterman (1980) ^c^	0	2	1	5	3	4

a Studies in descending order by year of publication.

b Black denotes the reference frame and blue denotes the alternate frame; one alternate codon position is underlined to show overlap with reference codon position 1 (e.g., position 3 for ss13).

c As reported by Lèbre and Gascuel (2017).

OLGenie estimates *d*_N_ and *d*_S_ in OLGs by modifying the method of Wei and Zhang (2015). Four expanded *d*_N_ and *d*_S_ measures are used: *d*_NN_, *d*_SN_, *d*_NS_, and *d*_SS_, where the first subscript refers to the reference gene and the second subscript refers to the alternate gene (NN, nonsynonymous/nonsynonymous; SN, synonymous/nonsynonymous; NS, nonsynonymous/synonymous; SS, synonymous/synonymous). For example, *d*_NS_ refers to the mean number of nucleotide substitutions per site that are nonsynonymous in the reference gene but synonymous in the alternate gene (NS). Given these values, *d*_N_/*d*_S_ may be estimated for the reference gene as *d*_NN_/*d*_SN_ or *d*_NS_/*d*_SS_, or for the alternate gene as *d*_NN_/*d*_NS_ or *d*_SN_/*d*_SS_. In each case, the effect of mutations in one of the two OLGs is held constant (N or S), ensuring a “fair comparison” in the other gene. For example, if nonsynonymous changes observed in the reference gene are disproportionately synonymous in the alternate gene (*d*_NS_ > *d*_NN_), the result will be *d*_NN_/*d*_NS_ < 1.0, and purifying selection on the alternate gene can be inferred (Hughes and Hughes 2005). In practice, *d*_NN_/*d*_NS_ rather than *d*_SN_/*d*_SS_ is typically used to test for selection in the alternate gene, as SS sites are usually too rare to allow a reliable estimate of *d*_SS_.

The original Wei–Zhang method is computationally prohibitive when many nucleotide variants are present in neighboring codons, and the size of the minimal bootstrap unit is data-dependent (Table 1). To circumvent these issues, we introduce three modifications: 1) consider each reference codon to be an independent unit of the alignment amenable to bootstrapping; 2) apply the Nei–Gojobori method to each OLG, as implemented in SNPGenie (Nei and Gojobori 1986; Nelson and Hughes 2015; Nelson et al. 2015); and 3) consider only single nucleotide differences, rather than all mutational pathways, that is, a given nucleotide change to a given codon either does (synonymous) or does not (nonsynonymous) encode the same amino acid. Modification (1) is not strictly true when two neighboring reference codons share sites with the same alternate codon, introducing biological nonindependence. Nevertheless, no individual site is included in more than one unit of the alignment, and the assumption of independence has proven widely effective (Nei and Kumar 2000), even though nearby codons may never evolve independently. Modification (3) is identical to the original Wei–Zhang method when a pair of sequences contains only one difference in contiguous codons. However, differences may be misclassified when ≥2 sites in contiguous codons differ. As a result, OLGenie tends to underestimate the denominator of *d*_N_/*d*_S_ (*d*_NS_ or *d*_SN_), biasing the ratio upward and yielding a conservative test of purifying selection that nevertheless has increased power over non-OLG *d*_N_/*d*_S_ (supplementary section S1, Supplementary Material online).

## Results and Discussion

### Assessment with Simulated Data

**Fig. 2:**
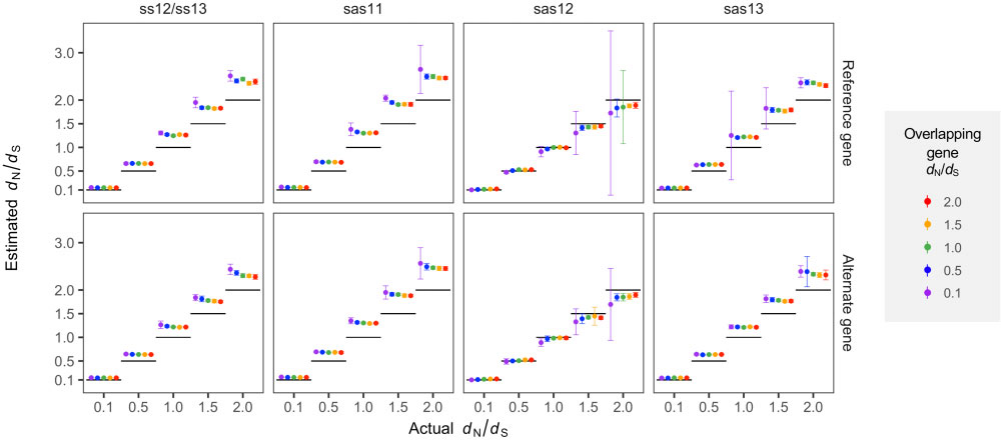
Assessment of OLGenie using simulated sequences. Calibration plots show the accuracy and precision of OLGenie *d*_N_/*d*_S_ estimates for the reference (top row; *d*_NN_/*d*_SN_) and alternate (bottom row; *d*_NN_/*d*_NS_) genes when mean pairwise distance is set to 0.0585 per site (median of biological controls). For each frame relationship, estimated *d*_N_/*d*_S_ is shown as a function of the actual simulated value, indicated by horizontal black line segments (*x* axis values), and of the *d*_N_/*d*_S_ value of the overlapping gene, indicated by color (left to right: purple = 0.1; blue = 0.5; green = 1.0; orange = 1.5; and red = 2.0). For example, all purple points in the top row refer to simulations with alternate gene *d*_N_/*d*_S_ = 0.1, whereas all purple points in the bottom row refer to simulations with reference gene *d*_N_/*d*_S_ = 0.1. To obtain highly accurate point estimates, each parameter combination (reference *d*_N_/*d*_S_, alternate *d*_N_/*d*_S_, frame) was simulated using 1,024 sequences of 100,000 codons (supplementary table S1, Supplementary Material online). Then, to obtain practical estimates of variance relevant to real OLG data, simulations were again carried out for each parameter combination so as to emulate our biological control data set: a sample size of 234, with sequence lengths (number of codons) and numbers of alleles (max 1,024) randomly sampled with replacement from the controls (supplementary table S2, Supplementary Material online). Error bars show SEM, estimated from replicates with defined *d*_N_/*d*_S_ values (≤234) using 10,000 bootstrap replicates (reference codon unit). A transition/transversion ratio (*R*) of 0.5 (equal rates) was used; similar results are obtained using *R *=* *2 (supplementary fig. S3, Supplementary Material online). Full simulation results are presented in supplementary tables S1–S6 and figures S1–S6, Supplementary Material online.

To evaluate OLGenie when selection dynamics are known, we first performed simulation experiments for each frame across a range of *d*_N_/*d*_S_ values, setting sequence divergence to that observed in our positive controls (median 0.0585; supplementary fig. S1, Supplementary Material online). Calibration plots reveal that OLGenie produces relatively accurate estimates, especially for purifying selection, improving substantially for lower sequence divergence (supplementary fig. S2, Supplementary Material online) and suffering minimally at higher transition/transversion ratios (supplementary fig. S3, Supplementary Material online). However, three biases are noteworthy: 1) except for frame sas12, *d*_N_/*d*_S_ is always overestimated; 2) except for sas12, *d*_N_/*d*_S_ overestimation increases when the OLG is under stronger purifying selection; and 3) for sas12, *d*_N_/*d*_S_ is somewhat underestimated for the OLG when *d*_N_/*d*_S_ ≥1 (fig. 2 and supplementary tables S1 and S2, Supplementary Material online). Bias (1) is mainly explained by modification (3) in the previous section. Bias (2) is explained by the failure to account for unobserved changes (multiple hits), for which no known correction is applicable to OLGs (Hughes et al. 2005); this causes the disproportionate underestimation of the denominator (*d*_NS_ or *d*_SN_) in the presence of purifying selection. Bias (3) may be due to the preponderance of “forbidden” codon combinations in sas12 (Lèbre and Gascuel 2017), which must necessarily be avoided to prevent STOP codons in the overlapping frame, leading to the overestimation of NN sites and underestimation of *d*_NN_. Additionally, our observations may be partly attributable to the fact that avoided STOP codons (TAA, TAG, and TGA) are AT-rich, implicitly favoring high GC content and biasing codon usage in OLGs (supplementary fig. S4 and table S3, Supplementary Material online) (Pavesi et al. 2018). Finally, for all frames, bias and variance for a given gene are highest when the alternate gene is under purifying selection.

Our simulations also allowed us to identify the most accurate and precise ratios for estimating each frame’s *d*_N_/*d*_S_. For ss12/ss13, sas11, and sas13, the rarest site class is SS (0–2.7% of sites), leading to high stochastic error when estimating *d*_SS_. Thus, for alternate genes in these frames, the *d*_NN_/*d*_NS_ ratio is relatively “site-rich” and preferred. Contrarily, for sas12, SS sites are usually more common (18.3%) than NS (7.4%) and SN (7.4%) sites, so that *d*_NN_/*d*_NS_ is preferred only 52.5% of the time (51.2–53.9%; binomial 95% C.I.) (supplementary tables S4 and S5 and figs. S5 and S6, Supplementary Material online). Thus, for alternate genes in sas12, either ratio can potentially be informative, and should be selected on a case-by-case basis, according to the number of sites: *d*_NN_/*d*_NS_ if the minimum of (NN, NS) ≫ minimum of (SN, SS); *d*_SN_/*d*_SS_ if the inequality is reversed; or both if the minima are approximately equal.

### Assessment with Biological Controls

**Fig. 3:**
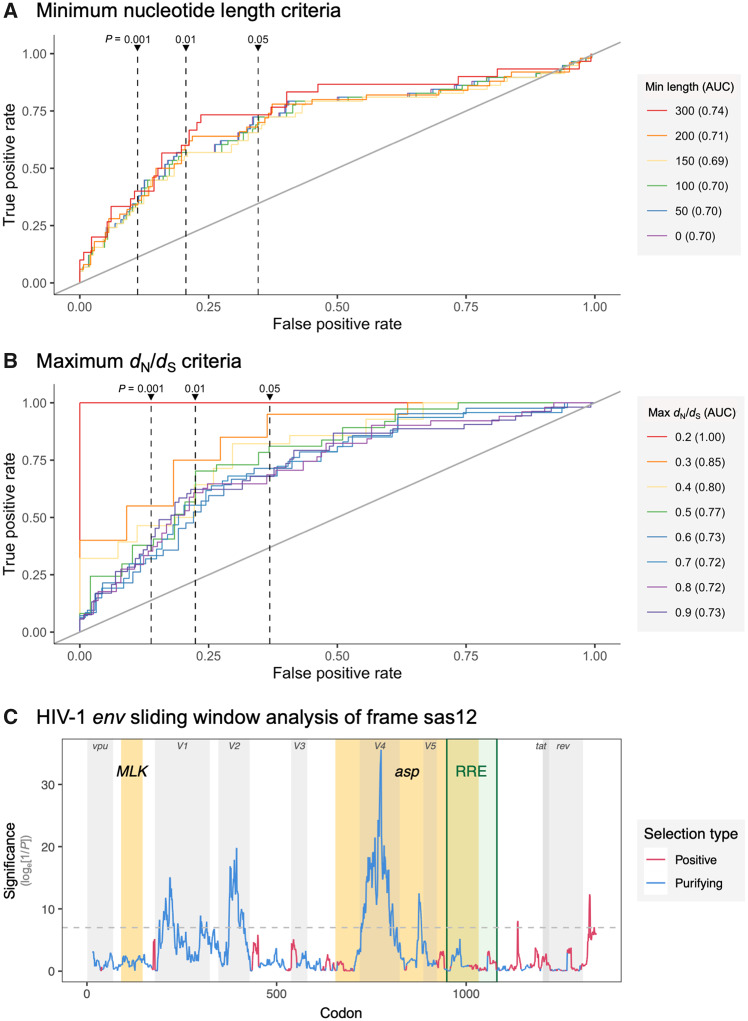
Assessment of OLGenie using biological controls. (*A* and *B*) Receiver operating characteristic (ROC) curves for overlapping (alternate) gene prediction at varying *P* value cut-offs. The *y* axis shows the true-positive rate (sensitivity) and the *x* axis shows the false-positive rate (1−specificity). Curves show subsets of the data corresponding to differing minimum length (*A*) and maximum *d*_N_/*d*_S_ (*B*) criteria, following the approach of Schlub et al. (2018), with red indicating the strictest criteria. The full data set is represented by purple in (*A*) (overlaps blue). Area under the curve (AUC) is reported in parentheses in the key (supplementary tables S6–S8, Supplementary Material online), and the ROC expected using random classification (AUC = 0.5) is shown as a diagonal gray line. Vertical dashed lines show mean false-positive rates for *P* value cut-offs of 0.001, 0.01, and 0.05 (left to right). The site-rich *d*_NN_/*d*_NS_ ratio was used to analyze 234 controls (81 ss12 and 153 ss13): 58 positive (16 ss12 and 42 ss13) and 176 negative (65 ss12 and 111 ss13). Of these, 162 (30 positive, 132 negative) had length ≥ 300 nt, and 14 (10 positive, 4 negative) had *d*_N_/*d*_S_ ≤0.2. (*C*) The HIV-1 *env* gene was analyzed in sas12 with the site-rich ratio *d*_NN_/*d*_NS_ using 25-codon sliding windows (step size = 1 codon), limiting to codons with ≥6 defined (nongap) sequences. The hypothesized *asp* gene is located at codons 655–1,033 (supplementary table S15, Supplementary Material online). The *y* axis shows significance, calculated as the natural logarithm of the inverse *P* value, as suggested by Firth (2014), using *Z* tests of the null hypothesis that *d*_NN_ = *d*_NS_ (1,000 bootstrap replicates per window; reference codon unit). The horizontal dashed gray line shows the multiple comparisons *P* value threshold (0.000924) suggested by Meydan et al. (2019) and described in supplementary section S5, Supplementary Material online, that is, a threshold of 0.05/(CDS length/window size). Results for other frames are shown in supplementary figure S9, Supplementary Material online. Positive selection (red) refers to *d*_N_/*d*_S_ > 1; purifying selection (blue) refers to *d*_N_/*d*_S_ < 1. Sequence features are described in supplementary table S15, Supplementary Material online and shown here as shaded rectangles: yellow for hypothesized sas12 genes, green for the highly structured RNA Rev response element (RRE), and gray otherwise.

To evaluate OLGenie’s performance with real biological data, we next applied the program to 58 known OLG (positive control) and 176 non-OLG (negative control) loci from viral genomes (Pavesi et al. 2018). Strict codon alignments were generated from quality-filtered BlastN hits (Materials and Methods). OLGenie results are 73% accurate (α = 0.05), with receiver operating characteristic curves yielding an area under the curve (AUC) of 0.70 for the full data set (supplementary table S6, Supplementary Material online). AUC increases marginally for longer sequences and drastically for lower *d*_N_/*d*_S_ values, reaching AUC = 1.0 for *d*_N_/*d*_S_ ≤ 0.2 (fig. 3 and supplementary tables S7 and S8, Supplementary Material online). Results are comparable even with less strict alignment criteria (supplementary figs. S7 and S8, tables S9–S12, and section S3, Supplementary Material online). Importantly, these results may underestimate OLGenie’s performance, as our data set included more negative than positive controls, and negative controls may include unannotated OLGs. For example, four negative controls of length 204–2,664 nt exhibit *d*_N_/*d*_S_ < 0.2, warranting investigation (supplementary table S6, Supplementary Material online). Finally, performance would likely improve for curated alignments limited to carefully defined taxonomic groups.

### Case Study: HIV-1’s Putative *Antisense Protein* Gene

Lastly, we examined the unresolved case of human immunodeficiency virus-1’s (HIV-1) *env*/*asp* sas12 overlap (Miller 1988; Torresilla et al. 2015), where the putative *antisense protein* (*asp*) gene has evaded detection by several bioinformatic methods, including non-OLG *d*_N_/*d*_S_ (Cassan et al. 2016; Schlub et al. 2018). We used OLGenie to test for purifying selection in three subregions of *env*: 1) 5′ non-OLG; 2) putative *asp*-encoding; and 3) 3′ non-OLG. Three data sets were used: 1) M group from Cassan et al. (2016) (1,723 codons × 23,831 sequences; functional *asp* hypothesized); 2) non-M groups from Cassan et al. (1,723 codons × 92 sequences; no functional *asp* hypothesized); and 3) HIV-1 BLAST hits for *env* using the same methods as our control data set (1,355 codons × 4,646 sequences). We employed *d*_NN_/*d*_NS_ for the alternate gene, as this ratio is by far the most site-rich for all *env* frames (i.e., sas12 site counts: NN = 2,127.2 and NS = 825.3, vs. SN = 190.1 and SS = 636.4; supplementary table S13, Supplementary Material online).

The sas12 *d*_N_/*d*_S_ ratio is significantly <1 in all three data sets for the 5′ non-OLG (*d*_N_/*d*_S_ ≤ 0.66; *P *=* *2.04 × 10^−7^) and *asp* (*d*_N_/*d*_S_ ≤ 0.58; *P *=* *2.75 × 10^−5^) subregions of *env*. The lowest ratio for each data set always occurs in *asp*, reaching very high significance in the BLAST data set (*d*_N_/*d*_S_ = 0.29; *P *=* *5.04 × 10^−25^). As a benchmark, our ss12/ss13 controls suggest a false-positive rate of 0% for *d*_N_/*d*_S_ ≤ 0.4 when employing *P *≤* *1.04 × 10^−6^ (based on 28 OLGs and 27 non-OLGs). The 3′ non-OLG region is also significant for the Cassan non-M groups (*d*_N_/*d*_S_ = 0.78, *P *=* *0.00921); however, the expected false-positive rate is high (∼22–28%) and the other two data sets are not significant in this region (*d*_N_/*d*_S_ ≥ 0.74; *P *≥* *0.107) (supplementary table S14, Supplementary Material online).

To test whether our results are an artifact of other sequence features, including the highly structured RNA Rev response element (RRE; supplementary table S15, Supplementary Material online; Fernandes et al. 2012), we also used OLGenie to perform sliding window analyses. Results show that purifying selection in the sas12 frame of *env* is most significant in regions of *asp* not overlapping the RRE (fig. 3*C*). The strongest evidence is observed in variable region 4, suggesting that accepted nonsynonymous changes in this region are disproportionately synonymous in *asp*. Significance is also attained in the correct frame for the two known ss12 OLGs, *vpu* and *rev* (supplementary figs. S9 and S10, Supplementary Material online). Thus, OLGenie specifically detects protein-coding function in all three data sets. Contrarily, Synplot2 shows the strongest evidence for synonymous constraint in the RRE, likely due to RNA structure rather than protein-coding function, and fails to detect *vpu* in the BLAST data set (supplementary fig. S11, Supplementary Material online). It should be noted that these OLGenie results concern the sas12 frame, for which the *d*_NN_/*d*_NS_ ratio is not always conservative (fig. 2), and that our biological controls were limited to the ss12 and ss13 frames. Nevertheless, our results provide evidence that purifying selection acts on the sas12 protein-coding frame of *env*, particularly in the *asp* region. This finding is corroborated by recent laboratory evidence demonstrating expression of ASP in multiple infected cell lines, where it localizes to both the host cell membrane and viral envelope upon activation of HIV-1 expression (Affram et al. 2019). This suggests ASP as a potential drug target, for which our sliding window results may be useful for identifying functionally constrained residues, that is, regions with low and highly significant *d*_N_/*d*_S_ (fig. 3*C* andsupplementary figs. S9 and S10 and supplementary data, Supplementary Material online).

## Conclusions

OLGenie provides a simple, accessible, and scalable method for estimating *d*_N_/*d*_S_ in OLGs. It utilizes a well-understood measure of natural selection that is specific to protein-coding genes, making it possible to directly compare functional constraint between OLGs and non-OLGs. Moreover, although its estimates of constraint are conservative, its discriminatory ability exceeds that of other methods (Schlub et al. 2018). Power is greatest at relatively low levels of sequence divergence, and may be increased in the future by incorporating mutational pathways or comparing conservative versus radical nonsynonymous changes. Even so, not all functional genes exhibit detectable selection, so that some OLGs are likely to be missed by any selection-based method. Nevertheless, because candidate OLGs are usually subject to costly downstream laboratory analyses, minimizing the false-positive rate is paramount. To this end, OLGenie achieves a false-positive rate of 0% for several subsets of our control data, for example, regions with *d*_N_/*d*_S_ < 0.4 and *P *≤* *1.04 × 10^−6^. OLGenie can therefore be used to predict OLG candidates with high confidence, allowing researchers to begin studying evolutionary evidence for OLGs at the genomic scale.

## Materials and Methods

OLGenie is written in Perl with no dependencies, and is freely available at https://github.com/chasewnelson/OLGenie (last accessed April 10, 2020). Estimates of *d* are obtained by calculating *d*_NN_ = *m*_NN_/*L*_NN_, *d*_SN_ = *m*_SN_/*L*_SN_, *d*_NS_ = *m*_NS_/*L*_NS_, or *d*_SS_ = *m*_SS_/*L*_SS_, where *m* is the mean number of differences and *L* is the mean number of sites between all allele pairs at each reference codon. Simulation scripts were modified from Wei and Zhang (2015). Biological control gene coordinates were obtained from Pavesi et al. (2018) and used to retrieve nucleotide sequences from the latest NCBI genome. Homologous sequences were obtained using BlastN (Altschul et al. 1990); excluded if they contained in-frame STOP codons or were <70% of query length (Hughes et al. 2005); translated using R Biostrings (Pagès et al. 2019); aligned using MAFFT v.7.150b (Katoh and Standley 2013); codon-aligned using PAL2NAL v14 (Suyama et al. 2006); and filtered to exclude redundant alleles. Only codon positions with ≥6 defined (nongap) sequences were used for estimating *d*_N_/*d*_S_ (Jordan and Goldman 2012). Statistical analyses were carried out in R v3.5.2 (R Core Team 2018). Significant deviations from *d*_N_*–d*_S_ = 0 were detected using *Z* tests after estimating the SE using 10,000 and 1,000 bootstrap replicates for genes and sliding windows, respectively (reference codon unit). Complete methods, results, and data are available in the Supplementary Material online and Zenodo at https://doi.org/10.5281/zenodo.3575391 (last accessed April 10, 2020).

## Supplementary Material

Supplementary data are available at *Molecular Biology and Evolution* online, with additional data available at Zenodo, https://doi.org/10.5281/zenodo.3575391 (last accessed April 10, 2020).

## Supplementary Material

msaa087_Supplementary_Data
