# Supplementary material for: OLGenie: Estimating Natural Selection to Predict Functional Overlapping Genes
Source: Mol Biol Evol. 2020 Apr 3;37(8):2440–9. doi: 10.1093/molbev/msaa087 (PMC7531306; doi:10.1093/molbev/msaa087)
Supplement: msaa087_Supplementary_Data [file msaa087_supplementary_data.zip › msaa087-suppl_data/Supplementary Text.pdf]

Supplementary Material

OLGenie: Estimating Natural Selection to Predict Functional Overlapping Genes

Chase W. Nelson,[1]* Zachary Ardern,[2,3] and Xinzhu Wei[4,5]

[1]Sackler Institute for Comparative Genomics, American Museum of Natural History, New York, New York, USA
[2]Chair for Microbial Ecology, Technische Universitaüt München, Freising, Germany
[3]ZIEL - Institute for Food & Health, Technische Universität München, Freising, Germany
[4]Department of Ecology and Evolutionary Biology, University of Michigan, Ann Arbor, MI, USA
[5]Department of Integrative Biology and Statistics, University of California, Berkeley, CA, USA

*Corresponding author: cnelson@amnh.org

Contents

S1. New Approaches 2

S2. Assessment with Simulated Data 3

S3. Assessment with Biological Controls 5

S4. Software, Statistics, and Visualization 8

S5. HIV-1 *env*/*asp* Sliding Window Analyses 9

Methods 9

Results and Discussion 10

OLGenie Analysis of BLAST Dataset in All Frames 10

OLGenie and Synplot2 Analyses of BLAST and Cassan Datasets 11

S6. References 13

S7. Supplementary Figures 15

S1. New Approaches

OLGenie analyzes a codon-based nucleotide alignment in nonamer (9nt) units, each of which is centered on a reference codon and includes a minimal overlapping unit of 6nt. Each 6nt unit contains one codon of the reference gene (*i.e.*, focal codon) and two partially overlapped codons of an alternate gene. The focal codon is located at sites 2-4 (ss12 and sas12) or 3-5 (ss13 and sas11) of the 6nt unit, where the remaining sites (1, 5, and 6 for ss12 and sas12; or 1, 2, and 6 for ss13 and sas11) constitute the 3 nucleotides for which the two flanking codons of the alternate gene do not overlap the focal codon (except for the sas13 frame; see main text, Figure 1). Once the coordinates of the minimal overlapping unit have been determined by the frame type, OLGenie examines each focal codon in the alignment, beginning with reference codon 2, and ending with the penultimate codon, as the first and last codons only partially overlap the alternate gene and therefore lack sufficient nucleotide context. Finally, for each focal codon, all alleles are compared to determine the number of NN (nonsynonymous-nonsynonymous), SN (synonymous-nonsynonymous), NS (nonsynonymous-synonymous), and SS (synonymous-synonymous) sites and differences. Estimates of nucleotide divergence are then obtained as $d_{NN} = m_{NN}/L_{NN}$, $d_{SN} = m_{SN}/L_{SN}$, $d_{NS} = m_{NS}/L_{NS}$, and $d_{SS} = m_{SS}/L_{SS}$, where m is the mean number of differences and L is the mean number of sites of each type for all pairs of sequences at each reference codon. The d_N/d_S ratio is then estimated as d_{NN}/d_{SN} or d_{NS}/d_{SS} for the reference gene, and as d_{NN}/d_{NS} or d_{SN}/d_{SS} for the alternate gene. For example, d_{NN}/d_{NS} quantifies the alternate gene's d_N/d_S by considering only mutations that are nonsynonymous in the reference gene (*i.e.*, the first subscripts are both N). In other words, if the proportion of nonsynonymous differences in the alternate gene which are also nonsynonymous in the reference gene (*i.e.*, NN differences) is smaller than expected under neutrality (*i.e.*, $d_{NN}/d_{NS} < 1.0$), then purifying selection on the alternate gene can be inferred.

We determined the best-performing (*i.e.*, most accurate and/or precise) ratio for each frame using our sets of ≤234 simulated sequences (genes) for each parameter set (Supplementary Table S2). This was done by identifying which ratio had the least absolute deviation from the actual (simulated) ratio and/or the lowest standard error in the majority of parameter combinations (`dNdS_*_deviation` and `dNdS_*_SE`, respectively, in Supplementary Table S5). For the reference gene, the best ratio was d_{NN}/d_{SN} for all ss12/ss13, sas11, and sas13 genes, and for 47% of sas12 genes. Complementarily, for the alternate gene, the best ratio was d_{NN}/d_{NS} for all ss12/ss13, sas11, and sas13 genes, and for 53% of sas12 genes (Figure 2; Supplementary Figures S2, S3, S5, S6; Supplementary Table S5). These results for ss12/ss13, sas11, and sas13 were expected because the better-performing ratios utilize the measure of d with the larger number of sites in the numerator or denominator, dubbed 'site-rich'. Indeed, only ~25% of sites are synonymous (Graur and Li 2000), making SS sites very rare in most frames. Thus, it makes sense to avoid the ratios that utilize SS sites (d_{SN}/d_{SS} and d_{NS}/d_{SS}), dubbed 'site-poor', because they will be subject to greater stochastic error. However, we also note that the accuracies are quite similar between the two ratios in many cases. Interestingly, sas12 is the only frame for which ratios employing d_{SS} are sometimes site-rich, and theoretical work also suggests that sas12 is unique in having the greatest number of amino acid constraints (Lèbre and Gascuel 2017). Although ratios employing d_{NN} in the numerator tend to be more accurate (Supplementary Table S5), ratios employing d_{SS} in the denominator can achieve lower variance if there are a sufficient number of both NS (reference), SN (alternate), and/or SS sites. It is also possible our

method could be extended in such cases by averaging the two ratios for each frame, potentially weighted by the number of sites comprising each.

When pairs of sequences exhibit ≥2 differences within the same or overlapping codons, the non-OLG Nei-Gojobori (Nei and Gojobori 1986), OLG Wei-Zhang (Wei and Zhang 2015), and other methods for estimating d_N/d_S consider all possible mutational pathways between sequences to determine the number of nonsynonymous and synonymous differences and sites. OLGenie simplifies analysis by determining only whether two alleles encode the same amino acid (1 synonymous difference) or do not encode the same amino acid (1 nonsynonymous difference). For pathways that involve both nonsynonymous and synonymous changes (*e.g.*, NS followed by SS), this approximation will misclassify synonymous differences (*e.g.*, SS differences) as nonsynonymous differences (*e.g.*, NS differences). For example, consider the sequence pair TC<u>AT</u>GG/TC<u>TA</u>GG with a reference frame beginning at site 1 and an alternate gene in frame ss13 (variable sites underlined). There are two possible mutational pathways, TCATGG→TCTTGG→TCTAGG and TCATGG→TCAAGG→TCTAGG, for a mean of 0.5 NN, 1 SN, and 0.5 NS differences. Instead of considering these pathways, OLGenie simply examines which amino acids are encoded by the corresponding codon in each frame, separately for each variable site. In this case, the first variable site occurs in a codon with no amino acid difference in the reference gene (Ser vs. Ser) but an amino acid difference in the alternate gene (His vs. Leu); thus, this difference is considered SN. The second variable site occurs in a codon with an amino acid difference in both genes (Trp vs. Arg and His vs. Leu); thus, this difference is considered NN. In this case, OLGenie's simplification resulted in 0.5 NS differences being misclassified as NN, for a total of 1 NN and 1 SN differences. Such misclassifications serve to underestimate d_S, the denominator of d_N/d_S, implying that the OLGenie method is biased toward high d_N/d_S values and constitutes a conservative test of purifying (negative) selection, with the possible exception of sas12 (Figure 2). In our simulations, in terms of magnitude, the worst overestimate for an alternate gene with the d_{NN}/d_{NS} ratio was d_N/d_S = 2.0 overestimated as 2.59 (sas11, reference gene d_N/d_S = 0.1) and the worst underestimate was d_N/d_S = 2.0 underestimated as 1.66 (sas12, reference gene d_N/d_S = 0.1) (Supplementary Table S1). Finally, OLGenie also excludes STOP codons and gaps from analysis (*e.g.*, for negative controls), such that their existence in either frame decreases the total number of sites considered but does not influence the d_N/d_S estimate.

S2. Assessment with Simulated Data

Simulations scripts were modified from Wei and Zhang (2015) to specify (1) d_N/d_S ratios of 0.1, 0.5, 1.0, 1.5, and 2.0 for each reference (`--dnds1`; ORF1) and alternate (`--dnds1`; ORF2) gene; (2) either 100,000 codons, or numbers of codons randomly sampled from our biological controls (`--num_codons`); (3) either 1,024 sequences, or numbers of sequences randomly sampled from our biological controls (`--num_seqs`; maximum of 1,024); (4) an emergent mean pairwise distance (*d*) of 0.0585 to match the median of our biological controls (`--distance_actual`); (5) a transition/transversion ratio of 0.5, 1, or 2 (`--R`); and (6) phylogenetically related sequences derived using a bifurcating tree.

With respect to (2), the code was first modified to remove STOP codons from the reference and alternate genes after sequence initiation, until none existed in either OLG. The code was then further modified to overestimate seed sequence length by a factor of 1.5 prior to STOP codon pruning, after which the remaining sequence length was trimmed to that requested by the user. The sorted codons lengths of our biological controls, from which our simulations sampled with replacement, were:

```
8, 16, 17, 18, 22, 23, 24, 28, 29, 33, 33, 33, 33, 36, 37, 37, 37, 41, 42, 42, 43, 44, 44,
45, 47, 50, 51, 51, 52, 53, 53, 53, 56, 57, 58, 62, 62, 62, 62, 64, 64, 66, 66, 66, 68, 68,
70, 70, 71, 72, 73, 73, 75, 77, 78, 80, 82, 84, 84, 84, 84, 85, 86, 87, 90, 91, 92, 96, 97,
98, 98, 98, 102, 104, 112, 114, 115, 115, 117, 119, 119, 120, 120, 122, 122, 126, 127, 127,
127, 130, 131, 132, 134, 135, 137, 138, 139, 140, 145, 148, 148, 148, 149, 150, 152, 154,
154, 159, 164, 166, 172, 174, 174, 176, 177, 179, 186, 187, 188, 189, 192, 194, 194, 197,
199, 202, 202, 202, 206, 206, 207, 213, 220, 222, 223, 228, 230, 231, 235, 235, 256, 282,
311, 313, 318, 318, 320, 322, 327, 334, 334, 347, 356, 366, 369, 370, 376, 376, 377, 387,
392, 395, 412, 417, 420, 420, 421, 421, 426, 457, 468, 473, 487, 493, 495, 503, 510, 523,
524, 524, 536, 548, 549, 549, 549, 550, 550, 564, 573, 574, 587, 616, 616, 620, 621, 622,
625, 630, 649, 666, 666, 696, 722, 764, 799, 817, 823, 839, 860, 886, 886, 892, 915, 980,
1046, 1118, 1216, 1232, 1246, 1472, 1472, 1581, 1607, 1677, 1692, 2107, 2108, 2146, 2182,
2182, 2227, 2688, 2791, 4369
```

With respect to (3), if the user requests fewer than 1,024 sequences, a simulation is first carried out with 1,024 sequences, after which the requested number is randomly sampled without replacement. The sequence lengths of our biological controls, from which our simulations sampled with replacement, were:

```
6, 6, 6, 6, 6, 7, 7, 7, 8, 8, 8, 8, 8, 8, 8, 9, 9, 9, 9, 9, 10, 10, 10, 11, 11, 11, 11, 11,
12, 13, 13, 13, 13, 14, 14, 14, 14, 14, 15, 15, 15, 15, 17, 18, 18, 19, 19, 20, 22, 22, 22,
23, 23, 24, 24, 24, 24, 24, 25, 25, 27, 27, 27, 28, 28, 29, 29, 29, 30, 31, 31, 31, 32, 32,
32, 32, 33, 33, 34, 34, 35, 35, 35, 35, 36, 37, 38, 38, 39, 39, 40, 40, 41, 42, 43, 43, 43,
44, 44, 45, 47, 47, 47, 48, 48, 48, 49, 49, 50, 51, 51, 54, 55, 57, 63, 64, 65, 66, 66, 67,
70, 70, 71, 71, 71, 72, 74, 75, 75, 76, 76, 77, 80, 81, 81, 82, 84, 85, 86, 86, 86, 87, 89,
90, 95, 98, 100, 107, 119, 120, 120, 126, 128, 133, 142, 144, 144, 144, 146, 147, 149, 149,
152, 155, 155, 159, 162, 179, 185, 194, 207, 207, 222, 223, 225, 230, 237, 240, 240, 267,
273, 275, 280, 290, 295, 301, 302, 302, 323, 332, 369, 381, 509, 509, 522, 661, 749, 749,
827, 833, 1064, 1107, 1177, 1313, 1360, 1619, 1872, 2747, 3476, 3706, 3748, 3748, 3832,
4778, 5208, 5663, 5671, 5671, 5833, 5833, 6012, 6161, 6812, 7247, 7247, 7306, 7744, 8149,
8820, 8944, 9167, 9954, 11026, 12206
```

With respect to (4), the original simulations produced values of *d* approximately equal to the distance requested by the user only under neutrality (`--dnds1` and `--dnds2` equal to 1), but lower under purifying selection and higher under positive selection. Thus, we empirically determined correction factors for the `--distance` parameter which would allow the user to specify the (approximate) emergent value of *d* desired at the end of the experiment, provided some combination of the aforementioned parameter values was used. This was achieved by first carrying out experiments with 100,000 codons using all parameter combinations with `--distance=0.05`, then using the highly accurate point estimates of emergent *d* to determine the correction factor which would result in the requested *d* under those settings. These values were hard-coded into the simulations, and are only expected to operate precisely if a combination of the exact parameter values above is requested (*e.g.*, `--dnds1=0.1`; `--dnds2=0.1`; and `--R=1`). After implementing this, emergent distances specified using a new distance option (`--

`distance_actual`) indeed fell in a narrow range from 0.0497 (sas12, R=0.5, reference d_N/d_S = alternate d_N/d_S = 0.1) to 0.0632 (sas12, R=1, reference d_N/d_S = alternate d_N/d_S = 2), approximately matching our biological controls. Nevertheless, there remained a slight but clear tendency for distance to increase with d_N/d_S (Supplementary Figure S1).

All simulation scripts are written in Perl and are available in the `supplementary_scripts` directory at the OLGenie GitHub page. The scripts should be called with 7 named arguments (required):

```
simulation_<frame_type>.pl \
--dnds1=<reference gene dN/dS> \
--dnds2=<alternate gene dN/dS> \
--num_codons=<number of codons> \
--num_seqs=<number of sequences> \
--distance=<mean pairwise differences per site> (original script) \
--distance_actual=<mean pairwise differences per site> (emergent) \
--R=<transition/transversion ratio> \
--outfile=<output file name or path>
```

For further details on the simulations, see Wei and Zhang (2015).

In addition to calibration for OLGenie, we also used our controls to investigate a possible relationship between frame, d_N/d_S, and GC content, independent of amino acid constraints. We predicted that mean GC content would exceed 50% due to the avoidance of (AT-rich) STOP codons within ORFs in general, and OLGs in particular. Indeed, mean GC content across all simulations ranged from 52.2 to 53.3% (Supplementary Figure S4). GC content did not vary substantially with d_N/d_S. However, we observed that GC content was always lowest (closest to 50%) for the sas12 frame. Thus, although theoretical work suggests that sas12 imposes the highest number of amino acid constraints on OLGs (Lèbre and Gascuel 2017), the avoidance of STOP codons apparently influences GC content less for sas12 than for any other frame. In summary, the presence of an ORF and the GC content of a sequence are not independent attributes, and our simulations suggest a GC content of ~53% is expected in OLGs, even in the absence of forces such as selection or mutational bias.

S3. Assessment with Biological Controls

Positive controls were obtained by extracting the coordinates of 80 OLGs from the file `journal.pone.0202513.s006.xls`, provided in the supplementary material of Pavesi et al. (2018), and downloading each reference nucleotide sequence from the latest NCBI genome/segment. Potentially homologous alleles were obtained using BLASTN (blastn suite > megablast; maximum of 200,000 target sequences; nucleotide collection = nr/nt database; expect threshold = 10; word size = 28; match score = 1; mismatch score = -2; gap cost = linear; query dates 07/08/2019-10/20/2019; https://blast.ncbi.nlm.nih.gov/Blast.cgi; Altschul et al. 1990). BLAST hit alignments were downloaded as XML files and processed manually in R studio using the commands in the scripts `xml2fasta.R` and `process_alignment.R` in the

`supplementary_scripts` directory at the OLGenie GitHub page (see in-line comments). Briefly, one copy of each unique sequence was retained (*i.e.*, unique alleles only), helping to obviate potential ascertainment biases, and only alignments with ≥2 unique alleles were used, as *d* is otherwise inestimable. BLAST hits were translated (R Biostrings::translate) and excluded if they (1) contained in-frame STOP codons, (2) were not a complete codon set (were not a multiple of 3), or (3) were <70% of query length (Hughes et al. 2005; Hughes and Friedman 2008). An exception to (1) was made if the reference genome/segment contained a STOP codon, in which case the codon was assumed to be a read-through and was allowed in every sequence. Translated sequences were aligned using MAFFT v.7.150b (Katoh and Standley 2013) with default settings, and codon alignments (CALs) were generated by imposing the amino acid alignment on the nucleotide sequences using PAL2NAL v14 (Suyama et al. 2006). Finally, when calculating d_N/d_S, codon positions were only included if they contained ≥6 defined (non-gap) sequences, a criterion recommended for d_N/d_S (Jordan and Goldman 2012; Jeffares et al. 2015).

After processing positive controls, negative controls were obtained by carrying out the same process for each non-overlapping (non-OLG) region of each viral genome/segment, *i.e.*, regions with no annotated OLG, and by analyzing those regions using the same frame as the positive control OLG from the same genome/segment (ss12 or ss13). If a genome/segment contained more than one OLG and the genes used different frames, its non-overlapping negative controls were each analyzed twice, once in each observed frame (ss12 and ss13). For HIV-1, the *asp* region was excluded (*i.e.*, considered neither a positive nor a negative control). Finally, after the above criteria were applied, we also required a genome/segment to have at least one remaining positive and one remaining negative control. These criteria retained 234 controls for analysis: 58 positive control OLGs (n = 16 ss12 and n = 42 ss13) and 176 negative control non-OLG regions (n = 65 ss12 and n = 111 ss13; Supplementary data, directory `controls_CAL`).

As an alternative to the strict codon alignment, we also analyzed our controls using two more lenient criteria. First, we applied the same procedure as the above, but did not require a minimum number of alleles at any codon position. This allowed the inclusion of controls from four additional viruses (flock house virus, hibiscus chlorotic ringspot virus, pea enation mosaic virus, and zaire ebolavirus), for which OLGenie performed well, with the result that AUC slightly improved (Supplementary Tables S9 and S10; Supplementary Figure S4; Supplementary data). Second, we analyzed the controls using a very simple procedure, requiring only (1) hit length identical to the query and (2) no STOP codons in the reference gene frame, unless also present in the reference sequence (script `process_alignment_simple.R` and in-line comments). AUC results again improved (Supplementary Tables S11 and S12; Supplementary Figure S5; Supplementary data, directory `controls_simple`)

After finalizing alignments, OLGenie was run on each positive and negative control using `OLGenie.pl` from the OLGenie GitHub page as follows, replacing 'ss13' with another frame when appropriate:

```
OLGenie.pl \
--fasta_file=seqs_DSS_noGap_noStop_fullCoverage_UNIQUE.fa \
--frame=ss13 \
--verbose
```

The results in each `OLGenie_codon_results.txt` results file were analyzed for significant deviations from d_N - d_S = 0 (null hypothesis of neutrality) with 10,000 bootstrap replicates (Nei and Kumar 2000) by employing the script `OLGenie_bootstrap.R` from the OLGenie GitHub page:

```
OLGenie_bootstrap.R \
OLGenie_codon_results.txt \
6 \
10000
```

where 6 refers to the minimum number of alleles required at each codon site, and 10000 refers to the number of bootstrap replicates per control. Bootstrap results for biological controls results shown in Figure 3A and 3B are available in Supplementary Table S6.

Receiver operating characteristic (ROC) curves were calculated manually in R Studio using the commands in the script `controls.R` in the `supplementary_scripts` directory at the OLGenie GitHub page (see in-line comments). Confusion matrix data, including true and false positive rates, were calculated for each of the following criteria: (1) each *P*-value observed in the dataset, with a region predicted to be an OLG if its *P*-value was less than the cut-off; (2) minimum lengths (L_{min}) of 0 (full dataset), 50, 100, 150, 200, and 300 nucleotides; and (3) maximum d_N/d_S values of 0.1, 0.2, 0.3, 0.4, 0.5, 0.6, 0.7, 0.8, 0.9, and Inf (no max). The area under the curve (AUC) was calculated using the R `diff()` (lagged differences) function, as described by Bruce and Bruce (2017). Conceptually, the AUC is a measure of how often (what proportion of the time) a true OLG (positive control) will be assigned a lower *P*-value than a false OLG (negative control). AUC = 0.8 is generally considered good classification ability (Spiegelhalter 2019). In our context, this value would imply that, if a true OLG and a negative control are both chosen at random, our test would assign a lower *P*-value to the true OLG 80% of the time.

When subsetting data by L_{min} or maximum d_N/d_S to calculate the ROC and AUC results, we followed the approach of Schlub et al. (2018) for continuity and comparison, *i.e.*, we considered the excluded data as 'not tested'. However, from the perspective of the initial dataset, the maximum possible sensitivity (true positive rate) is greatly reduced by such filtering. For example, if a filtering criterion excludes 50% of true positives, the maximum possible sensitivity (true positive rate) might be considered 50% and the ROC might end at the point [1,0.5], *i.e.*, 50% of true positives are excluded from the test 'by definition'. Indeed, in our dataset, only 10 positive control OLG sequences (17.2% of true positives) remained when requiring $d_N/d_S \leq 0.2$ (AUC = 1.0); similarly, in the Schlub et al. dataset, only 199 OLG sequences (20.8%) remained when requiring L_{min} = 300nt (AUC = 0.89). This highlights that these methods are able to achieve a very low false positive rate — usually the priority when candidates are being selected for costly downstream laboratory analysis — but that this is sometimes done at the expense of a large fraction of the data.

All control scripts and data are available in the `supplementary_scripts` directory at the OLGenie GitHub page or in the Supplementary Material. Results are reported in the following directory structure:

```
/controls/<frame_type>/<virus>/<region_ID>/<results_files>
```

Here, `<frame_type>` is ss12 or ss13; `<virus>` is the name of the virus reported by Pavesi et al. (2018); and `<region_ID>` begins with "OL" (overlapping; positive control) or "NOL" (non-overlapping; negative control), followed by the name of one (NOL) or two (OL) genes, followed by the nucleotide coordinates of the region in the genome/segment. For example, results for the ORF3/ORF4 overlap in tobacco bushy top virus are reported in the directory `/controls/ss13/tobacco_bushy_top_virus/OL_ORF3_ORF4_2772-3470/`. Finally, up to 10 files (`<results_files>`) are present within each `<region_ID>` directory:

1. `sequence.fasta`, the query sequence, obtained from the NCBI reference genome.
2. `sequence_rev_compl.fasta`, present only for examples with reference genes on the antisense strand, in which case this file contains the reverse complement sequence that was used as the BLAST query.
3. `<RID>-Alignment.xml`, the BLAST results of the query sequence.
4. `<RID>-Alignment.fasta`, the output of `xml2fasta.R`, which contains a multi-FASTA file of (unaligned) BLAST hits. Note that, despite the name, the sequences in the file `<RID>-Alignment.fasta` do not form an alignment, as they are simply the raw sequences extracted from the BLAST XML file. Hits with length <70% the query are subsequently eliminated by filtering in `process_alignment.R`.
5. `seqs_uniq_goodName_filtered_DSS.fa`, a FASTA format DNA String Set (DSS) created by `process_alignment.R`, which contains the quality-filtered nucleotide alleles.
6. `seqs_uniq_goodName_filtered_AASS.fa`, a FASTA format Amino Acid String Set (AASS) created by `process_alignment.R`, which contains the amino acid translations of the sequences in (4).
7. `seqs_uniq_goodName_filtered_AASS_PAL.fa`, a MAFFT alignment of the amino acid sequences in (5).
8. `seqs_uniq_goodName_filtered_DSS_CAL.fa`, a PAL2NAL codon alignment of the nucleotide sequences in (4), created by imposing the amino acid alignment present in (6), used as the input for `OLGenie.pl`.
9. `OLGenie_codon_results.txt`, the output of `OLGenie.pl`.
10. `OLGenie_bootstrap_results.txt`, the output of `OLGenie_bootstrap.R`.

S4. Software, Statistics, and Visualization

The OLGenie software, supplementary scripts, and example data are freely available online at the OLGenie GitHub page https://github.com/chasewnelson/OLGenie. All statistical analyses were carried out in R v3.5.2 (R Core Team 2018), employing the following additional packages and their dependencies: `Biostrings`, `boot`, `caTools`, and `tidyverse`. Figures were created

in R using the packages `ggplot2`, `patchwork`, `RColorBrewer`, and `scales`, and modified in Microsoft PowerPoint.

S5. HIV-1 *env*/*asp* Sliding Window Analyses

Methods

Codon alignments (CALs) of HIV-1 *env* BLAST hits were constructed using the same procedure applied to our controls, limiting to *bona fide* HIV-1 sequences by excluding hits with headers containing the expressions "[Ss]imian", "[Ss]ynthetic construct", [Cc]loning", or "T-cell" (see above, Assessment with Biological Controls, strict codon alignment with a minimum of 6 alleles). The *env* query sequence was obtained from HIV-1 reference genome NC_001802.1, nucleotide sites 5771-8341. Cassan M group and non-M groups alignments were obtained from the supplementary material of Cassan et al. (2016), files `envnuclM.fasta` and `envnuclnonM.fasta`. All three HIV-1 datasets were then analyzed using both OLGenie and Synplot2 (Firth 2014; http://www.firthlab.path.cam.ac.uk/index.html).

For OLGenie, we first analyzed three separate regions of *env*: (1) 5′ non-OLG (codons 1-654 in the BLAST alignment and codons 1-849 in the Cassan alignments), (2) putative *asp*-encoding (codons 655-1,033 in the BLAST alignment and codons 850-1,275 in the Cassan alignments); and (3) 3′-non-OLG (codons 1,034-1,354 in the BLAST alignment and codons 1,276-1,722 in the Cassan alignments). Next, to obtain sliding window results comparable to Synplot2, d_N/d_S was estimated in sliding windows of 25 codons (step size of 1 codon), and *P*-values were obtained using a *Z*-test of the null hypothesis that $d_N - d_S = 0$, using 1,000 bootstrap replicates to estimate the standard error for each window. For inclusion in the d_N/d_S estimate, we again required a codon position to have ≥6 defined alleles. Windows were excluded if the the d_N/d_S or standard error were undefined (NA).

For Synplot2, each alignment was analyzed after editing the maximum number of sequence pairs permitted to 100,000 and recompiling the program as instructed in the Synplot2 README file. Phylogenetic trees for each nucleotide alignment were reconstructed using FastTree 2 v2.1.10 SSE3 (Price et al. 2010; http://www.microbesonline.org/fasttree/) with default settings. Sequence pairs tracing around the tree were obtained by extracting sequence names in the order provided in the Newick file. The “plot” output file was then used to calculate *P*-values for sliding windows of 25 codons (step size of 1 codon) as the normal distribution percentile corresponding to the appropriate *Z* value using the following R command:

```
pnorm(runmean(subs_observed - subs_expected, 25) /
      sqrt(runmean(subs_stdev * subs_stdev, 25)) * sqrt(25))
```

where `subs_observed` and `subs_expected` are the observed and expected numbers of synonymous substitutions, respectively; `subs_stdev` is the standard error of the number of synonymous substitutions; and 25 is the sliding window size in codons.

For both Synplot2 and OLGenie, sliding window plots were constructed by displaying significance as log(1/*P*), where log is the natural logarithm and *P* is the *P*-value reported by each respective method, with results shown in Supplementary Figures S6-S8. Horizontal grey lines shows the multiple comparisons correction suggested for Synplot2 by Meydan et al. (2019), *i.e.*, a *P*-value threshold of 0.05 / (*CDS length* / *window size*), where *CDS length* and *window size* are measured in codons. Other sequence features are also shown, with coordinates listed in Supplementary Table S15.

All scripts and data used in the HIV-1 *env* analyses are available in the `supplementary_scripts` directory at the OLGenie GitHub or the Supplementary Material, with full sliding window results in the directory `/HIV1_env/`. Commands used to analyze sliding windows are reported in the script `sliding_windows.R` (see in-line comments).

Results and Discussion

OLGenie Analysis of BLAST Dataset in All Frames

First, to gain a comprehensive understanding of the d_N/d_S ratio's behavior in HIV-1 *env*, we used the BLAST dataset to analyze all frames using the site-rich ratios d_{NN}/d_{SN} (reference) and d_{NN}/d_{NS} (alternate) (Supplementary Table S13). Results are shown in Supplementary Figure S9. Sliding windows revealed peaks of significant purifying selection in both the reference and alternate frames in the context of several frame relationships. In the reference frame, the highest peaks occurred (*env*, 5′ to 3′) near (1) the putative *MLK*-encoding region (Berger et al. 2015) in the contexts of ss12, ss13, sas11, and sas13; (2) an OLG-free region between V2 and V3 in the contexts of ss12, ss13, sas11, and sas13; (3) the putative stop codon of *asp* in the context of sas13; and (4) an OLG-free region between *RRE* and *rev* in the contexts of ss12, ss13, sas11, and sas13. In the alternate frame, the highest peaks occurred near (1) *asp* in its hypothesized context, sas12, exceeding any purifying selection seen elsewhere in any frame; (2) V1 and V2 in the contexts of ss12, sas11, and sas12; and (3) *rev* in its true context, ss12. The OLG *vpu* also attained marginal significance in its true context, ss12.

With respect to positive selection, in the reference frame, the variable (V) regions (particularly V3) are known to encode important immune epitopes (Holmes et al. 1992; Seibert et al. 1995; Hughes 1999). However, significant $d_N/d_S > 1$ was not attained in V3, instead appearing in V1 and V4 (ss13 and sas13). It is noteworthy that the peak of positive selection in V4 overlaps a fraction of the peak of purifying selection in *asp*. Focusing instead on the alternate frame, several studies suggest that sas12 encodes proteins which induce an immune response, including *asp*, *MLK*, and others (Berger et al. 2015; Bet et al. 2015; Liu et al. 2019). However, significant positive selection was only attained in V1 and *rev* (sas13). It is interesting to note that OLGenie tends to overestimate d_N/d_S for all frames except sas12 when using the d_{NN}/d_{NS} ratio (Figure 2). Thus, these results for sas12 may be legitimate, even though results indicating positive selection in other frames should usually be interpreted with caution. For example, even if the the sas13 frame of the *rev* region does not encode protein, an artifactual signal of positive selection could result from purifying selection on ss12 (the true context of *rev*). Furthermore, we note that transcription, translation, and positive selection for immune escape can occur even when a protein product

plays no biological role, such that positive selection is a more dubious indicator of functionality than purifying selection. Finally, even if two OLGs experience purifying selection, it is likely that neither amino acid sequence can be fully optimized without compromising the other, leading to a relatively weak signal. This may be one reason that OLGs are thought to be more common in smaller genomes with high mutation rates, where genomic data compression is relatively important. Indeed, functional regions are 'segregated' between the known OLGs in HIV-1 (Fernandes et al. 2016).

Our sliding window results may explain why the *env* locus, and *asp* in particular, has been so recalcitrant to study by other methods. Besides the fact that sas12 is the most enigmatic frame, *env* also encodes myriad overlapping features, and a mixture of positive and purifying selection can dilute signals of functionality. Our results also suggest that *a priori* knowledge of the correct frame may be useful, as *bona fide* signal may be limited to the correct frame, whereas spurious signal may be shared by multiple frames. For example, it is interesting that evidence for *MLK* function disappears in both the reference and alternate frames when measured in its purported context, sas12. We also note that *asp* utilizes a frame relationship (sas12) that differs from that of all positive controls (ss12/ss13). Knowledge of the correct frame could be gained by first limiting to ORFs, or first filtering candidates by a method to detect long ORFs, such as that of Schlub et al. (2018). Future work is needed to understand the biological factors that influence the differences between frame contexts. For example, for *rev*, how does legitimate purifying selection on ss12 affect the d_N/d_S ratio in the context of sas13? Finally, although not the case for *env*, more work is needed to understand the behavior of the two d_N/d_S ratio estimators when both ratios are similarly site-rich.

OLGenie and Synplot2 Analyses of BLAST and Cassan Datasets

We next analyzed two datasets from Cassan et al. (2016): the M group (functional *asp* hypothesized) and the non-M groups (no functional *asp* hypothesized). One notable aspect of these data is that the major (most common) allele is undefined (---) for ~51% of codon sites in both the M and non-M groups, and the vast majority of nonamers are incompletely defined (*i.e.*, contain one or more gaps). This may present a problem, as codon sites with few defined alleles may strongly influence downstream analyses, and d_N/d_S estimates should ideally be based on sites with ≥6 defined alleles (Jordan and Goldman 2012). Moreover, if these gaps are indicative of alignment errors, this could lead to failure to detect purifying selection or spurious detection of positive selection (Schneider et al. 2009). Thus, because previous analyses of the Cassan data with Synplot2 presumably used all codon sites, we used all sites for our analyses of the Cassan datasets, but limited to sites with ≥6 defined alleles for both our Synplot2 and OLGenie analyses of the BLAST dataset. As confident multiple sequence alignments of OLGs are currently lacking, further work is needed to obtain high-quality alignments of this and other OLGs.

First, we employed OLGenie to analyze the Cassan datasets using the same sliding window procedure implemented for the BLAST dataset, limiting to sas12. Results are shown in Supplementary Figure S10. The strongest peak of purifying selection overlaps V1 in the M group sequences, dwarfing a similar spike in the BLAST dataset. The *asp* region shows two peaks of purifying selection: one overlapping V3 that is shared by the M and non-M groups, and one occurring before V5 that is specific to the M group. Both peaks are also apparent in the BLAST

dataset (Figure 3C). Finally, another peak also overlaps V2 in both the M and non-M groups, also apparent in the BLAST dataset. Evidence for *MLK* functionality is again lacking.

The Synplot2 analysis of Cassan et al. (2016) showed evidence for synonymous constraint only at sites of *asp* that overlap *RRE*, probably not indicative of protein-coding function. On the other hand, OLGenie shows significant purifying selection only at sites outside *RRE*. To compare the performances of OLGenie and Synplot2 for the BLAST dataset and to replicate the Cassan et al. results for their own dataset, we employed Synplot2 to all three datasets, masking (-) sites with <6 defined alleles so the BLAST results could be compared between methods. Results are shown in Supplementary Figure S11. For every dataset, unlike OLGenie but consistent with the Synplot2 analysis Cassan et al. (2016), our Synplot2 results show the greatest evidence for synonymous constraint in the RRE region of *asp*, likely as a result of purifying selection on RNA structure rather than protein-coding function. Finally, unlike OLGenie (Supplementary Figure S9), Synplot2 does not detect *vpu* in the BLAST dataset.

S6. References

Altschul SF, Gish W, Miller W, Myers EW, Lipman DJ. 1990. Basic Local Alignment Search Tool. Journal of Molecular Biology 215:403–410.

Berger CT, Llano A, Carlson JM, Brumme ZL, Brockman MA, Cedeño S, Harrigan PR, Kaufmann DE, Heckerman D, Meyerhans A, et al. 2015. Immune Screening Identifies Novel T Cell Targets Encoded by Antisense Reading Frames of HIV-1. J. Virol. 89:4015–4019.

Bet A, Maze E, Bansal A, Sterrett S, Gross A, Graff-Dubois S, Samri A, Guihot A, Katlama C, Theodorou I, et al. 2015. The HIV-1 Antisense Protein (ASP) induces CD8 T cell responses during chronic infection. Retrovirology 12:15.

Bruce P, Bruce A. 2017. Practical Statistics for Data Science. First Edition. Sebastopol, CA: O'Reilly

Cassan E, Arigon-Chifolleau A-M, Mesnard J-M, Gross A, Gascuel O. 2016. Concomitant emergence of the antisense protein gene of HIV-1 and of the pandemic. Proc Natl Acad Sci USA 113:11537–11542.

Fernandes JD, Faust TB, Strauli NB, Smith C, Crosby DC, Nakamura RL, Hernandez RD, Frankel AD. 2016. Functional Segregation of Overlapping Genes in HIV. Cell 167:1762–1773.

Firth AE. 2014. Mapping overlapping functional elements embedded within the protein-coding regions of RNA viruses. Nucleic Acids Research 42:12425–12439.

Graur D, Li W-H. 2000. Fundamentals of Molecular Evolution. Second Edition. Sunderland, MA: Sinauer Associates, Inc. Publishers

Hughes AL, Ekollu V, Friedman R, Rose JR. 2005. Gene Family Content-Based Phylogeny of Prokaryotes: The Effect of Criteria for Inferring Homology. Systematic Biology 54:268–276.

Hughes AL, Friedman R. 2008. Codon-based tests of positive selection, branch lengths, and the evolution of mammalian immune system genes. Immunogenetics 60:495–506.

Jeffares DC, Tomiczek B, Sojo V, dos Reis M. 2015. A beginners guide to estimating the non-synonymous to synonymous rate ratio of all protein-coding genes in a genome. In: Peacock C, editor. Parasite Genomics Protocols. Vol. 1201. Methods in Molecular Biology. New York: Springer. p. 65–90. Available from: http://dx.doi.org/10.1007/978-1-4939-1438-8_4

Jordan G, Goldman N. 2012. The Effects of Alignment Error and Alignment Filtering on the Sitewise Detection of Positive Selection. Molecular Biology and Evolution 29:1125–1139.

Katoh K, Standley DM. 2013. MAFFT Multiple Sequence Alignment Software Version 7: Improvements in Performance and Usability. Molecular Biology and Evolution 30:772–780.

Lèbre S, Gascuel O. 2017. The combinatorics of overlapping genes. Journal of Theoretical Biology 415:90–101.

Liu Z, Torresilla C, Xiao Y, Nguyen PT, Caté C, Barbosa K, Rassart É, Cen S, Bourgault S, Barbeau B. 2019. HIV-1 Antisense Protein of Different Clades Induces Autophagy and Associates with the Autophagy Factor p62. J Virol 93:e01757-18.

Meydan S, Marks J, Klepacki D, Sharma V, Baranov PV, Firth AE, Margus T, Kefi A, Vázquez-Laslop N, Mankin AS. 2019. Retapamulin-Assisted Ribosome Profiling Reveals the Alternative Bacterial Proteome. Molecular Cell 74:481–493.

Nei M, Gojobori T. 1986. Simple methods for estimating the numbers of synonymous and nonsynonymous nucleotide substitutions. Molecular Biology and Evolution 3:418–426.

Nei M, Kumar S. 2000. Molecular Evolution and Phylogenetics. New York, NY: Oxford University Press

Pavesi A, Vianelli A, Chirico N, Bao Y, Blinkova O, Belshaw R, Firth A, Karlin D. 2018. Overlapping genes and the proteins they encode differ significantly in their sequence composition from non-overlapping genes. PLoS ONE 13:e0202513.
Price MN, Dehal PS, Arkin AP. 2010. FastTree 2 – Approximately Maximum-Likelihood Trees for Large Alignments.Poon AFY, editor. PLoS ONE 5:e9490.
R Core Team. 2018. R: A language and environment for statistical computing. Vienna, Austria: R Foundation for Statistical Computing Available from: https://www.R-project.org/
Schlub TE, Buchmann JP, Holmes EC. 2018. A simple method to detect candidate overlapping genes in viruses using single genome sequences. Molecular Biology and Evolution 35:2572–2581.
Schneider A, Souvorov A, Sabath N, Landan G, Gonnet GH, Graur D. 2009. Estimates of Positive Darwinian Selection Are Inflated by Errors in Sequencing, Annotation, and Alignment. Genome Biology and Evolution 1:114–118.
Spiegelhalter D. 2019. The Art of Statistics. Great Britain: Pelican
Suyama M, Torrents D, Bork P. 2006. PAL2NAL: robust conversion of protein sequence alignments into the corresponding codon alignments. Nucleic Acids Research 34:W609–W612.
Wei X, Zhang J. 2015. A Simple Method for Estimating the Strength of Natural Selection on Overlapping Genes. Genome Biology and Evolution 7:381–390.

S7. Supplementary Figures

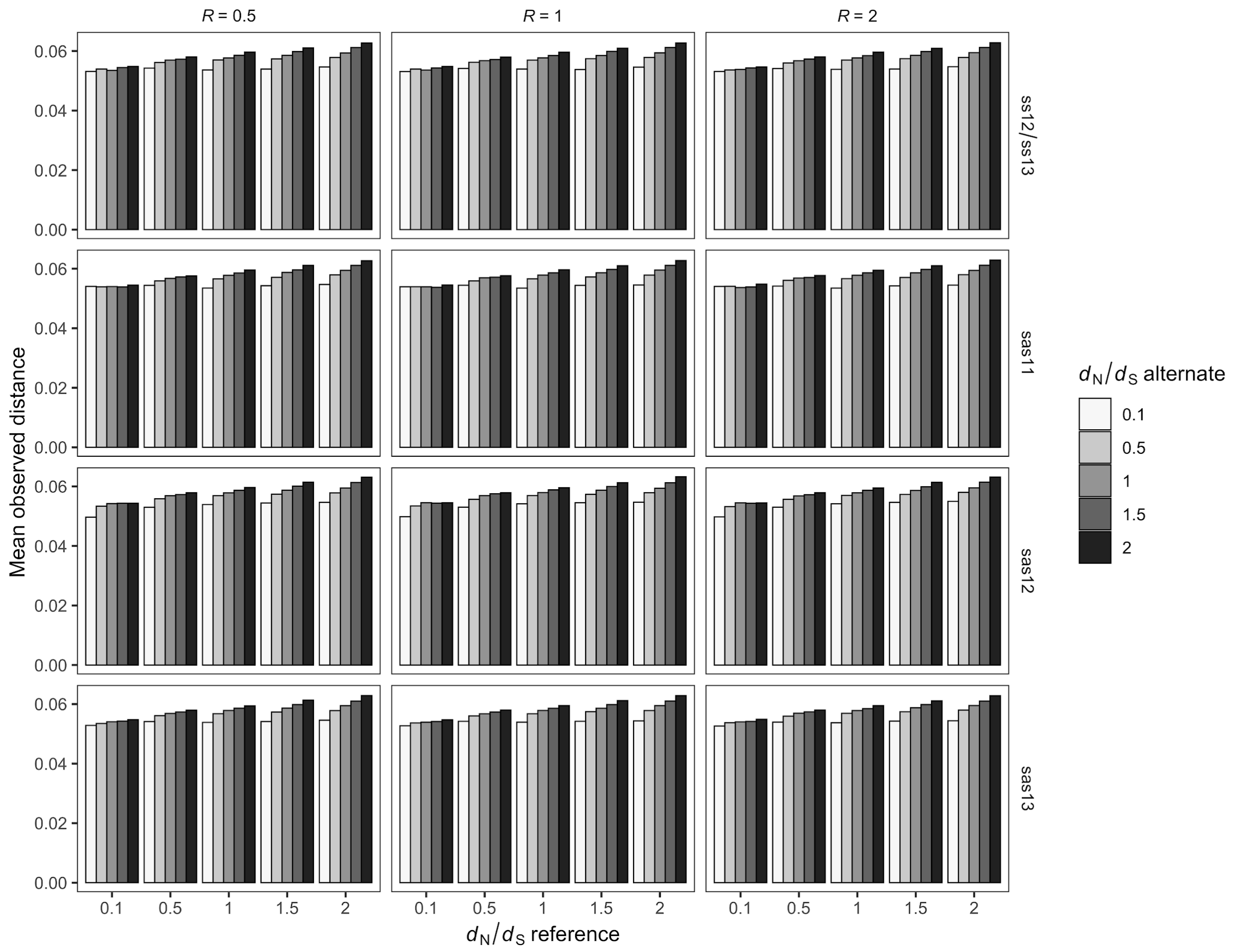

Supplementary Figure S1. Mean sequence divergence (*d* = differences per site) among simulated sequences at varying d_N/d_S and transition/transversion ratio (*R*) values. The main text shows results for *R* = 0.5 (Figure 1). To match our biological positive controls (median *d* = 0.0585), the argument `--distance_actual=0.0585` was used with each simulation script (https://github.com/chasewnelson/OLGenie/supplementary_scripts). The simulations produced slightly higher distances for higher d_N/d_S values, but the range was small, from a minimum of 0.0497 (sas12, *R* = 0.5, d_N/d_S reference = 0.1, d_N/d_S alternate = 0.1) to a maximum of 0.0632 (sas12, *R* = 1, d_N/d_S reference = 2, d_N/d_S alternate = 2).

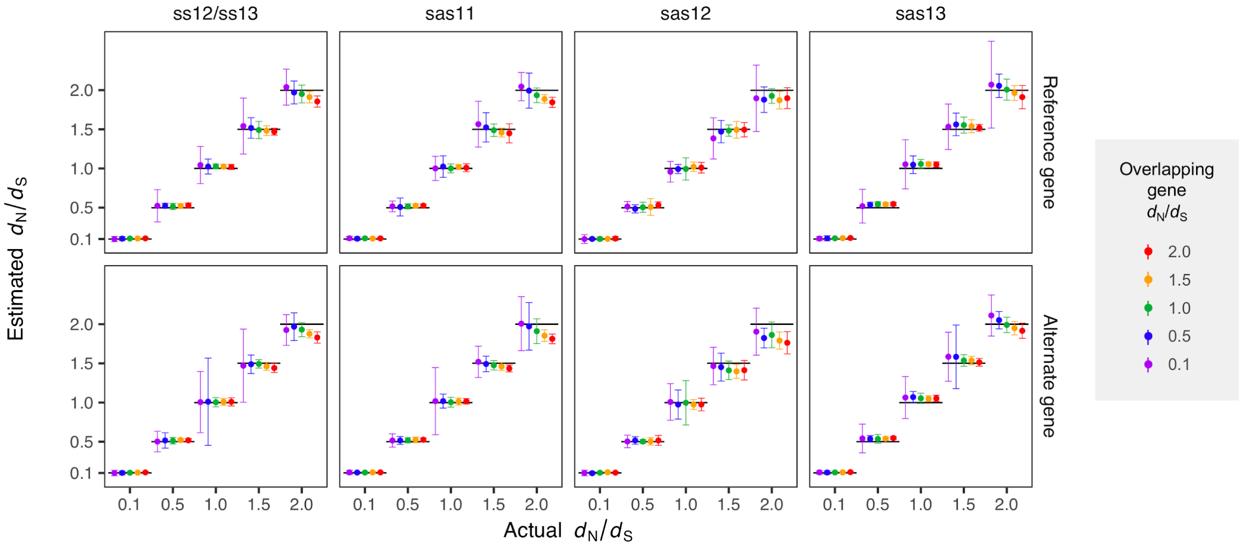

Supplementary Figure S2. Calibration plots for site-rich ratios using the unmodified simulations of Wei and Zhang (2015) with the argument `--distance=0.05`. Emergent mean sequence distances varied greatly with d_N/d_S, ranging from 0.0046 (d_N/d_S reference=0.1, d_N/d_S alternate=0.1) to 0.125 (d_N/d_S reference=2, d_N/d_S alternate=2) (compare to distances or ~0.0585, main text, Figure 2). For ss12/ss13 and sas13, the site-rich ratios were always d_{NN}/d_{SN} (reference) and d_{NN}/d_{NS} (alternate). For sas12, the site-rich ratio varied on a case-by-case basis.

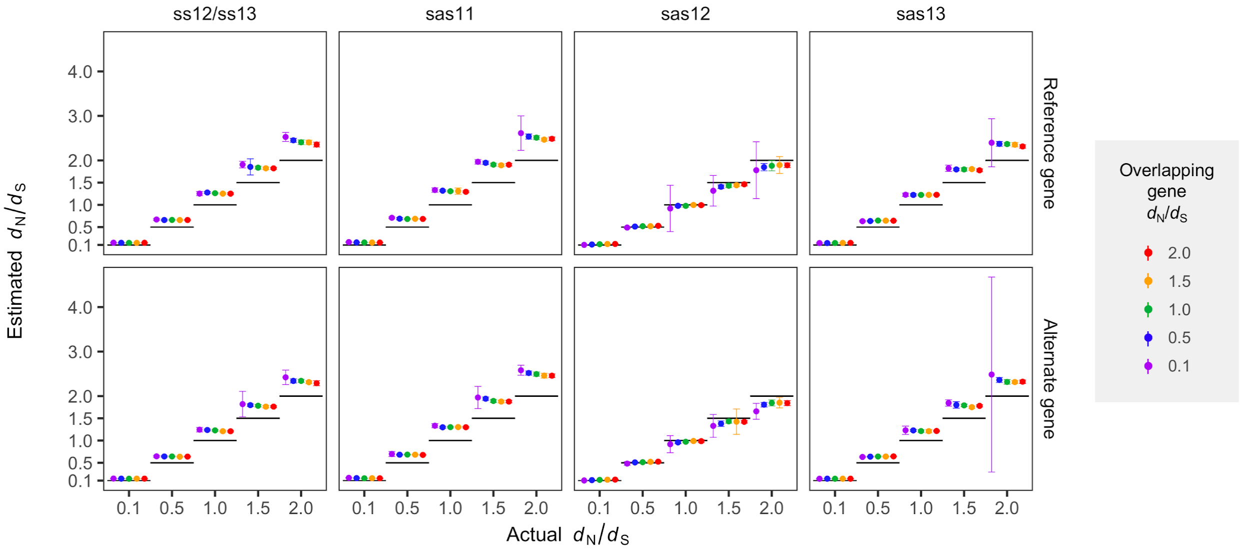

Supplementary Figure S3. Calibration plots with an increased transition/transversion ratio (R) of 2 for d_{NN}/d_{SN} (reference) and d_{NN}/d_{NS} (alternate) ratios when sequence divergence was ~0.0585 (compare to R=0.5 in Figure 2).

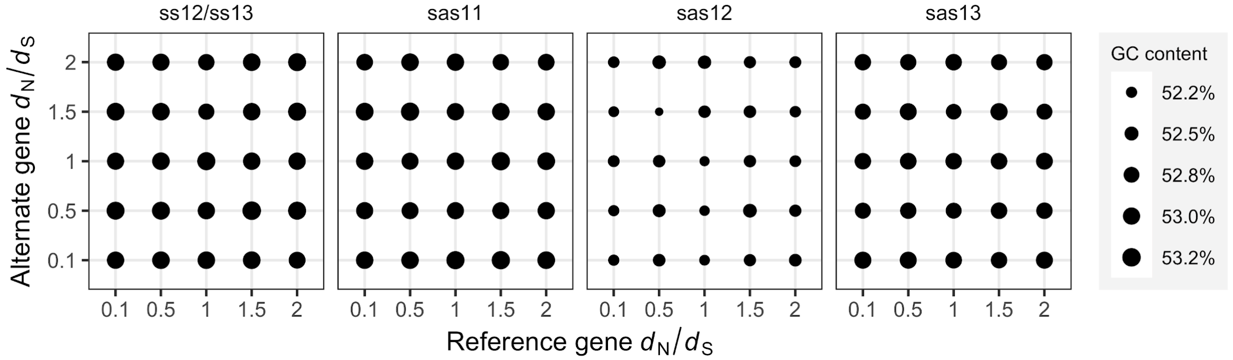

Supplementary Figure S4. GC content in overlapping genes as a function of frame (ss12/ss13, sas11, sas12, and sas13) and d_N/d_S (0.1 to 2.0, reference and alternate genes). Estimates are based on the 100,000-codon simulations used to derive the d_N/d_S point estimates in Figure 2 of the main text. To maximize visual discriminatory ability, larger dots correspond to higher GC content; however, note that point estimates of mean GC content fall in a very narrow range from 52.2% (sas12, d_N/d_S reference = 0.5, d_N/d_S alternate = 1.5) to 53.3% (ss12/ss13, reference d_N/d_S = 1.5, alternate d_N/d_S = 0.5). Expected GC content was always lowest for the sas12 frame. Full results are reported in Supplementary Table S3.

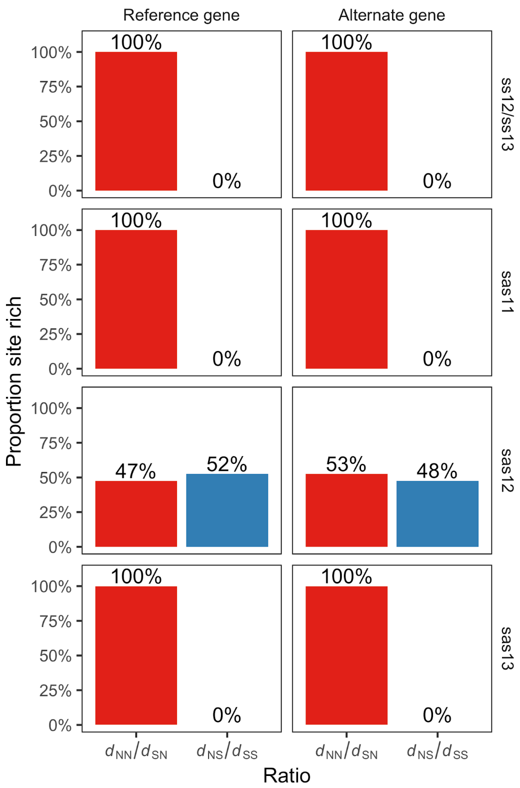

Supplementary Figure S5. Expected proportion of the time a particular ratio is site-rich for genes in a given frame in our simulated sequences. For frames ss12/ss13, sas11, and sas13, ratios employing d_{NN} in the numerator were always site-rich; indeed, sas11 never contained any SS sites (Supplementary Table S5). For sas12, ratios employing d_{NN} in the numerator or d_{SS} in the denominator were each site-rich about half the time. Thus, when analyzing genes in sas12, the site-rich ratio depends on the specific gene sequence and should be identified on a case-by-case basis.

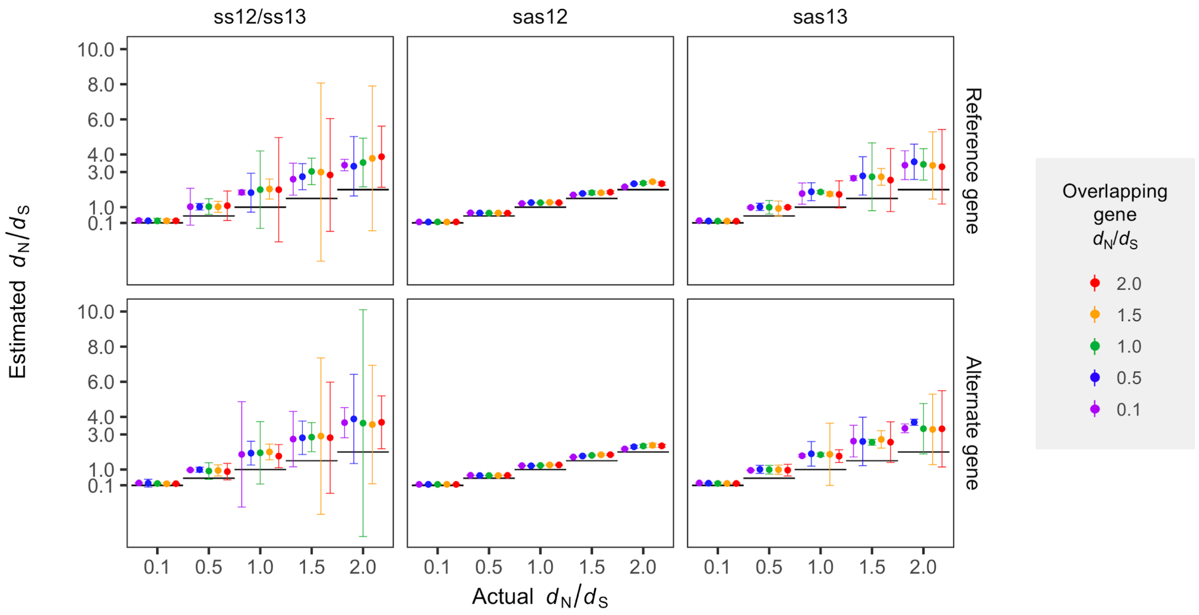

Supplementary Figure S6. Calibration plots for ratios employing d_{SS} in the denominator for the reference (d_{NS}/d_{SS}; top row) and alternate (d_{SN}/d_{SS}; bottom row) genes. No results are shown for sas11 because this frame never contained SS sites. Compare to results using ratios employing d_{NN} in the numerator in the main text (Figure 2), noting the differing y axes.

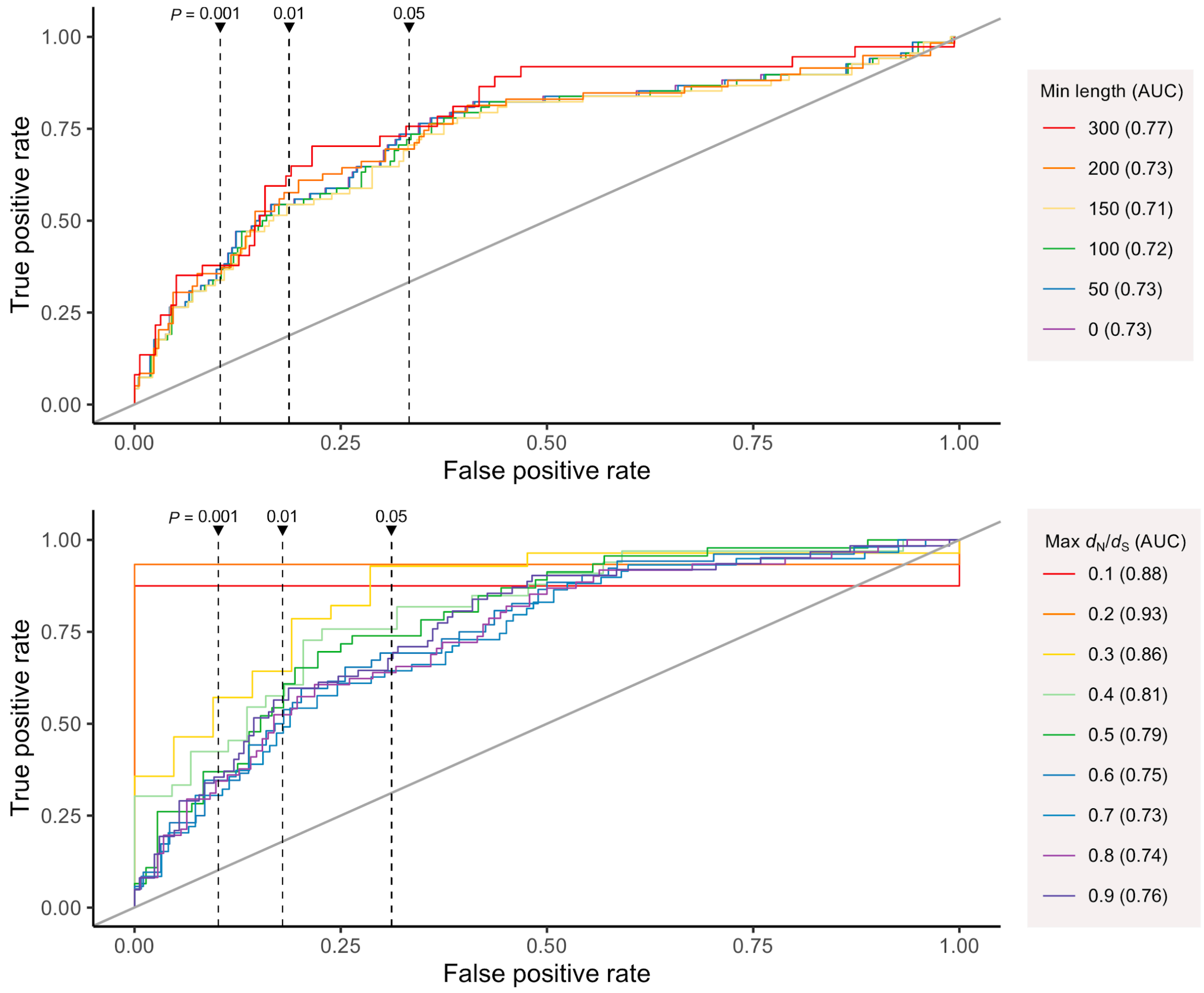

Supplementary Figure S7. Receiver operating characteristic (ROC) curves using biological controls to estimate OLGenie's true positive (y-axis; sensitivity) and false positive (x-axis) rates using a strict codon alignment with no minimum allele criterion (compare to main text Figure 3, which uses a minimum 6 alleles). Curves were plotted for subsets of the data corresponding to minimum length (*A*) and maximum d_N/d_S (*B*) criteria, following the ROC approach of Schlub et al. (2018). ROC (confusion matrix) and AUC results are presented in Supplementary Tables S9 and S10.

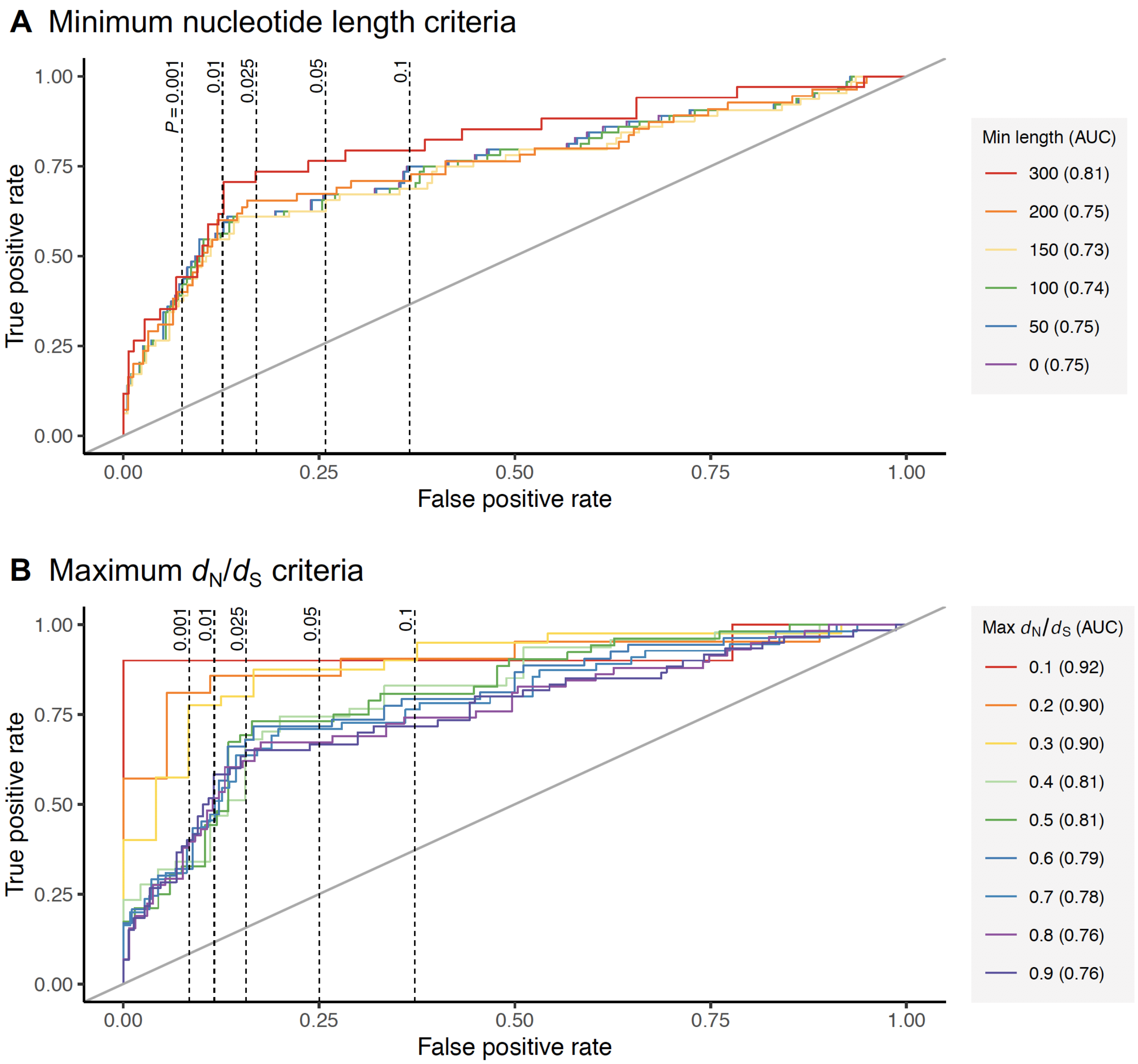

Supplementary Figure S8. Receiver operating characteristic (ROC) curves using biological controls to estimate OLGenie's true positive (y-axis; sensitivity) and false positive (x-axis) rates using simple criteria: sequence length matches query and no in-frame STOP codons (compare to main text Figure 3, which is based on a strict codon alignment with a minimum of 6 alleles per codon position). Curves were plotted for subsets of the data corresponding to minimum length (*A*) and maximum d_N/d_S (*B*) criteria, following the ROC approach of Schlub et al. (2018). ROC (confusion matrix) and AUC results are presented in Supplementary Tables S11 and S12.

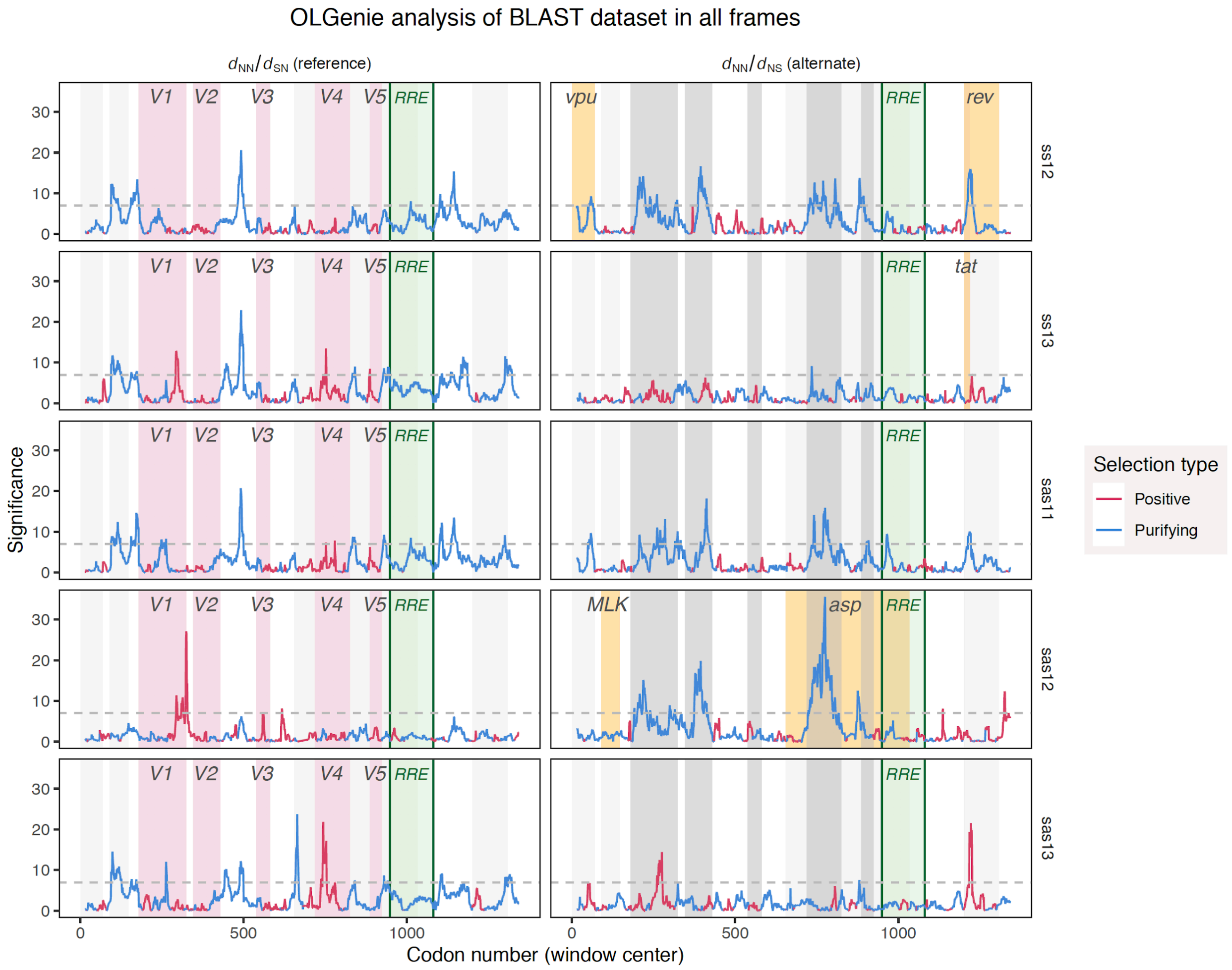

Supplementary Figure S9. OLGenie sliding window d_N/d_S analysis of the HIV-1 *env* BLAST dataset (alignment length=1,355 codons) using site-rich ratios for the reference (left; d_{NN}/d_{SN}) and alternate (right; d_{NN}/d_{NS}) frames (window size=25 codons; step size=1 codon). Selection significance (y axis) was measured as the natural logarithm of the inverse *P*-value, calculated using *Z*-tests (1,000 bootstrap replicates per window). The horizontal dashed grey line shows the multiple comparisons *P*-value threshold described in the Supplementary text, corresponding to a *P*-value of 0.000924. Windows with undefined d_N/d_S values or standard errors were excluded. Positive selection (red) refers to $d_N > d_S$; purifying selection (blue) refers to $d_N < d_S$. Sequence features of *env* are listed in Supplementary Table S15 and shown here as shaded rectangles: red for variable (V) regions expected to be under positive selection in the reference (*env*) frame; yellow for OLGs expected to be under purifying selection in an alternate frame; and green for the RNA Rev response element (RRE). Features are here labeled and colored only in their known (or hypothesized) contexts, and are grey in non-encoding frame(s) for point of reference.

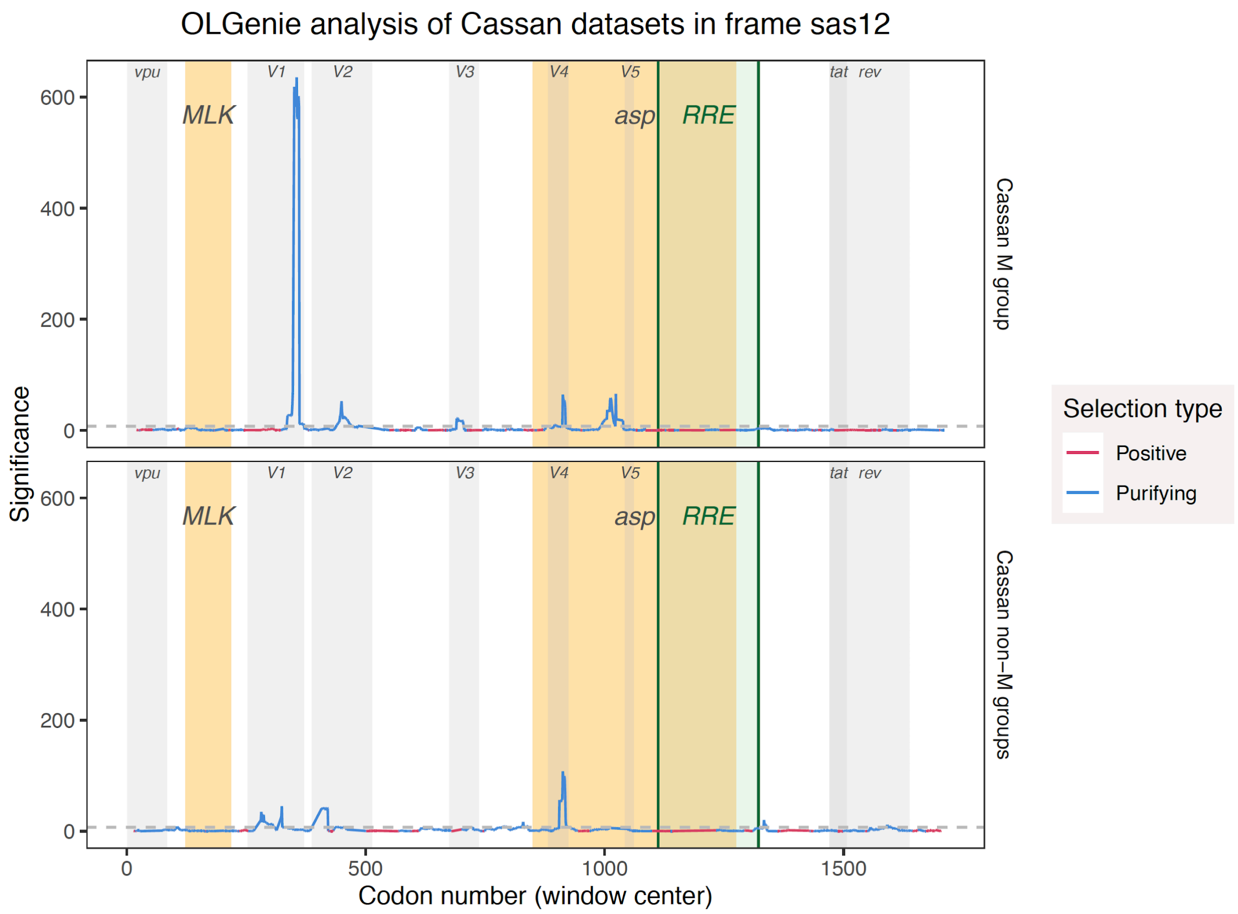

Supplementary Figure S10. OLGenie sliding window d_N/d_S analysis of the HIV-1 *env* Cassan datasets (alignment length=1,723 codons) using the site-rich ratio d_{NN}/d_{NS} for the alternate frame sas12, using the same procedure applied to the BLAST dataset (minimum codons=6; window size=25 codons; step size=1 codon). Selection significance (y axis) was measured as the natural logarithm of the inverse *P*-value, calculated using *Z*-tests (1,000 bootstrap replicates per window). The horizontal dashed grey line shows the multiple comparisons *P*-value threshold described in the Supplementary text, corresponding to a *P*-value of 0.000726. Windows with undefined d_N/d_S values or standard errors were excluded. Positive selection (red) refers to $d_N > d_S$; purifying selection (blue) refers to $d_N < d_S$. Sequence features of *env* are listed in Supplementary Table S15 and shown here as shaded rectangles: yellow for OLGs expected to be under purifying selection in the sas12 frame; green for the RNA Rev response element (RRE); and grey otherwise.

Synplot2 analysis of all three datasets

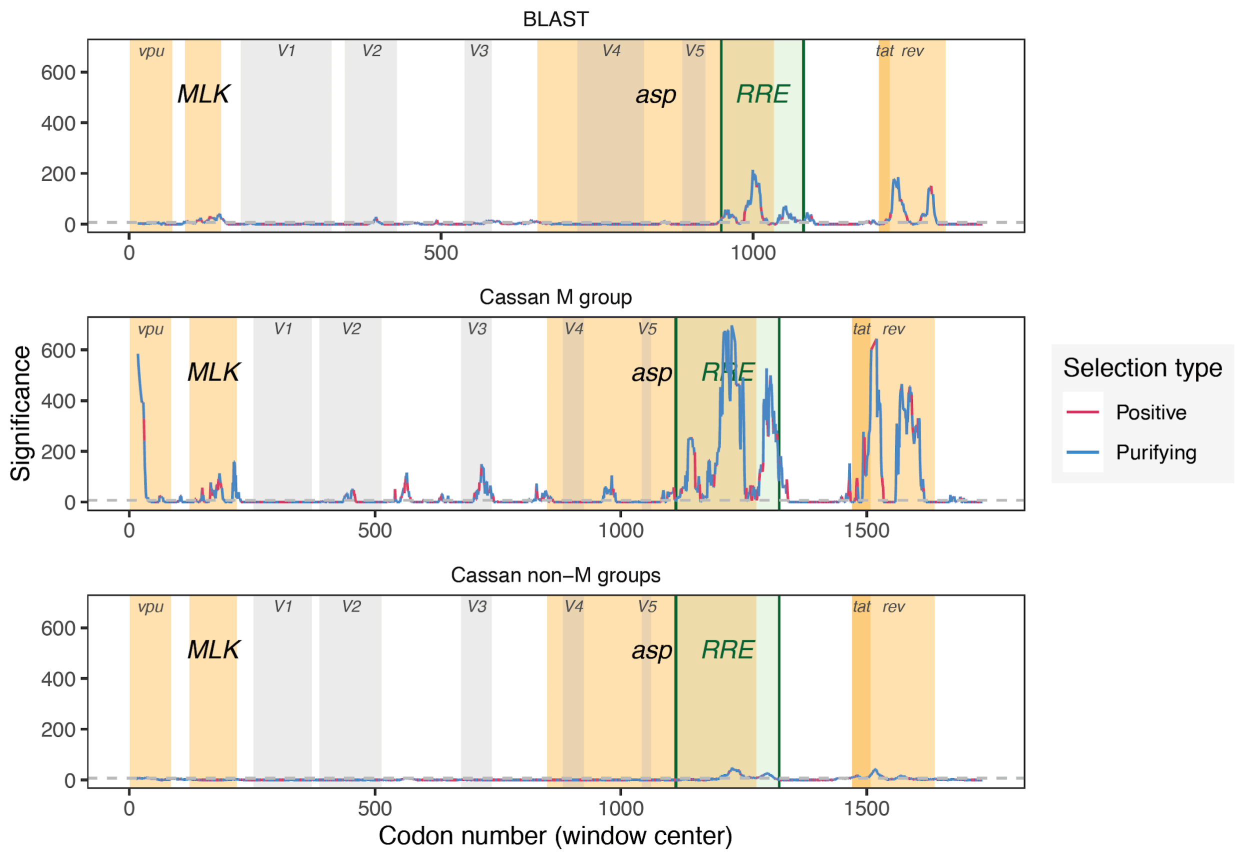

Supplementary Figure S11. Synplot2 sliding window analyses of all three HIV-1 *env* dataset: BLAST, Cassan M group (functional *asp* hypothesized), and Cassan non-M groups (no functional *asp* hypothesized) (window size=25 codons; step size=1 codon). Note that the x axes differ for the BLAST dataset (alignment length=1,355 codons) and the Cassan datasets (alignment length=1,723 codons). For straightforward comparison to OLGenie analyses, a minimum of 6 codons was required per codon site for the BLAST dataset. Note that Synplot2 implicitly tests for both protein- and non-protein-coding constraint in all overlapping frames. Selection significance (y axis) was measured as the natural logarithm of the inverse *P*-value, calculated using *Z*-tests (for OLGenie, 1,000 bootstrap replicates per window; for Synplot2, the analytic formula suggested by the Synplot2 README file; Firth 2014). The horizontal dashed grey line shows the multiple comparisons *P*-value threshold described in the Supplementary text, corresponding to *P*-values of 0.000923 (BLAST) and 0.000725 (Cassan). Positive selection (red) refers to more than expected substitutions; purifying selection (blue) refers to fewer than expected substitutions. Sequence features of *env* are listed in Supplementary Table S15 and shown here as shaded rectangles: yellow for OLGs expected to be under purifying selection in the sas12 frame; green for the RNA Rev response element (RRE); and grey otherwise.
